# The Association Between Cholesterol, High-Density Lipoprotein, and Glucose Index and Mortality in Young and Middle-Aged Adults With Diabetes or Prediabetes: NHANES Data (1999–2018)

**DOI:** 10.14740/cr2190

**Published:** 2026-04-15

**Authors:** Chuan Bo He, Yong Zeng, Liang Zhang

**Affiliations:** a Department of Cardiology, Daxing Hospital, Capital Medical University, Beijing, China; b Department of Cardiology, Beijing Anzhen Hospital, Capital Medical University, Beijing Institute of Heart, Lung and Blood Vessel Disease, Beijing, China

**Keywords:** Cholesterol, High-density lipoprotein, Glucose index, Mortality, Diabetes, Prediabetes, NHANES

## Abstract

**Background:**

This study aimed to investigate the association between cholesterol, high-density lipoprotein, and glucose (CHG) index and mortality among patients with diabetes or prediabetes and to determine whether this association changes with age.

**Methods:**

From the National Health and Nutrition Examination Survey (1999–2018), 14,369 patients with diabetes or prediabetes were selected and divided into two age groups: 50 years and younger, and older than 50 years. The Cox proportional hazards models, restricted cubic spline (RCS) models and interaction test were employed to analyze the associations between CHG index and mortality.

**Results:**

During a median follow-up of 96 months, 2,741 deaths from all causes and 899 deaths related to cardiovascular issues were recorded. Cox proportional hazards regression analysis found a positive correlation between the CHG index and mortality from all causes, as well as cardiovascular causes. According to the RCS model, there is a U-shaped correlation between the baseline CHG index and mortality, and age significantly interacts with this relationship. The study revealed a significant correlation between increased CHG and a heightened risk of death in people aged 50 years and below.

**Conclusions:**

We found that the CHG index is associated with mortality in individuals younger than 50 years, underscoring the critical role of the CHG index in identifying and screening for mortality risk among those with early-onset diabetes.

## Introduction

Diabetes and prediabetes represent significant public health challenges worldwide, primarily due to their strong association with cardiovascular disease (CVD). Statistics from the International Diabetes Federation indicate that over 537 million adults around the world had diabetes in 2021, with expectations for this number to grow [1]. Significantly, the number of young individuals with diabetes is rising globally. Data from the International Diabetes Federation’s 2021 diabetes map indicate that nearly 20% of people aged 20 to 40 worldwide have diabetes, with more than 90% of cases being type 2 diabetes mellitus (T2DM) [2]. Therefore, detecting high-risk population and implementing effective primary prevention strategies is critical for reducing mortality.

Multiple studies have shown that hyperglycemia and insulin resistance (IR) are key factors contributing to CV complications, such as myocardial infarction (MI), stroke, and peripheral artery disease [3]. IR is closely linked to various abnormalities associated with metabolic syndrome, including obesity and dyslipidemia. Collectively, these factors collectively increase the risk of coronary heart disease [4]. Studies indicate that IR is closely associated with the onset of CVD, especially during the prediabetes stage, where its presence notably increases the risk of CVD [5, 6]. In individuals with diabetes, lipid abnormalities are closely associated with an increased risk of atherosclerotic CVD [7]. In the context of prediabetes, a higher prevalence of dyslipidemia is typically observed, which may further exacerbate the risk of atherosclerosis [8]. In addition, individuals with prediabetes and diabetes should have lipid management approaches that specifically target the phenotype with high triglycerides (TGs) and low high-density lipoprotein cholesterol (HDL-C), given its connection to a heightened risk of CVD [9].

Recently, the cholesterol, high-density lipoprotein, and glucose (CHG) index, a combined measure of cholesterol and glucose, has been introduced as a method for diagnosing type 2 diabetes by assessing IR [10]. Research has shown that a higher CHG index is significantly associated with a heightened risk of CVD among middle-aged people [11]. The predictive power of the CHG index in evaluating CVD risk is similar to that of the triglyceride-glucose (TyG) index, according to the available data. The prognostic value of the CHG index among the general population with diabetes, particularly across different age strata, has not been explored. The objective of the current study was to: 1) investigate the association between the baseline CHG index and all-cause and cardiovascular mortality in adults with diabetes or prediabetes; 2) determine whether this association is modified by age, with a specific focus on younger versus older adults.

## Materials and Methods

### Study cohort and design

**Figure 1:**
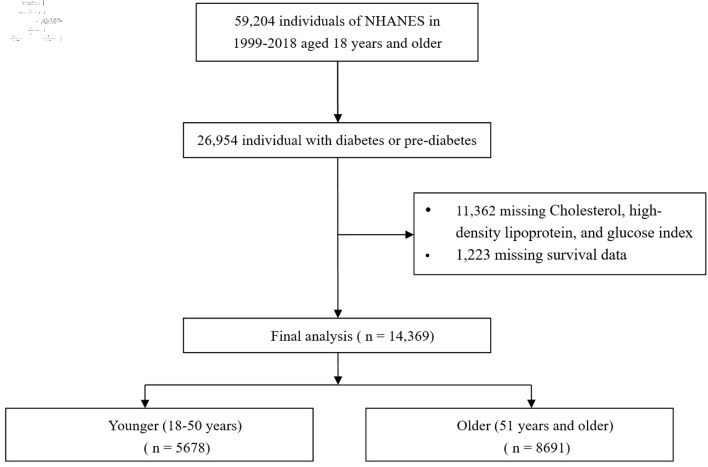
Flow chart of study participants. NHANES: National Health and Nutrition Examination Survey.

The National Health and Nutrition Examination Survey (NHANES) constitutes a critical research program aimed at evaluating the health and nutritional profiles of adults and children across the United States. Conducted by the Centers for Disease Control and Prevention (CDC), NHANES generates vital health metrics that guide public health policies and scientific investigations. The survey’s procedures have received approval from the Research Ethics Review Board of the National Center for Health Statistics (NCHS). To ensure the protection of participant rights, written informed consent was secured from all individuals taking part in the study. Furthermore, the datasets employed and examined in this research are publicly available through the official NHANES website. In this analysis, data from NHANES spanning 1999 to 2018 were extracted, and the study did not need the Institutional Review Board (IRB) approval. The ethics statement was not applicable to this study. Based on the diagnostic guidelines set forth by the American Diabetes Association (ADA), diabetes is defined as a self-reported diagnosis, the administration of insulin or oral hypoglycemic medications, fasting blood glucose (FBG) levels equal to or exceeding 126 mg/dL, or hemoglobin A1c (HbA1c) levels at or above 6.5%. Prediabetes is identified by a self-reported prediabetes condition, FBG levels ranging from 100 mg/dL to 125 mg/dL, or HbA1c values between 5.7% and 6.4% [12]. A cohort of 26,954 adults diagnosed with either diabetes or prediabetes was assessed (including individuals aged 18 to 85 years). Following the exclusion of participants lacking data for the CHG index (n = 11,362), all-cause mortality dates, or baseline medical conditions (n = 1,223), a total of 14,369 participants were ultimately incorporated into the present study (Fig. 1).

### Assessment of covariates

Data pertaining to a comprehensive set of demographic and health-related variables were obtained via household interviews conducted as part of the NHANES. These variables included age, sex, race/ethnicity, educational background, household income, smoking history, existing medical conditions, and prescription drug use. Body mass index (BMI) was derived by dividing an individual’s weight in kilograms by the square of their height in meters. For analytical purposes, race/ethnicity was segmented into four distinct classifications: White, Black, Mexican, or other. Educational attainment was organized into a three-tiered system: less than a high school diploma, high school graduate or equivalent, and college-level education or higher. Smoking behavior was documented using three classifications: never smoker, former smoker, or current smoker. Alcohol intake was stratified into four categories: heavy drinker (characterized by consuming three or more drinks daily for women or four or more for men, or participating in binge drinking (defined as four or more drinks on one occasion for women or five or more for men) on five or more days per month), moderate drinker (defined as two or more drinks daily for women or three or more for men, or engaging in binge drinking on two or more days monthly), mild drinker (individuals not meeting the criteria for heavy or moderate drinking), and nondrinker. Furthermore, a series of clinical biomarkers—including fasting plasma glucose, HbA1c, TGs, total cholesterol (TC), low-density lipoprotein cholesterol (LDL-C), and HDL-C—were quantified through laboratory analyses performed within the NHANES framework.

### Assessment of CHG index

The CHG index was determined using the formula: CHG index = Ln (TC (mg/dL) × FBG (mg/dL)/2 × HDL (mg/dL)). Participants were divided into four groups (Q1, Q2, Q3, Q4) based on the quartiles of the CHG index, with the Q1 group serving as the reference.

### Ascertainment of mortality

The mortality status of the follow-up cohort was determined using the NHANES public-use linked mortality file, which was current as of December 31, 2019. A probabilistic matching algorithm, overseen by the National Center for Health Statistics (NCHS), was used to integrate this dataset with the National Death Index (NDI). Moreover, the International Statistical Classification of Diseases, 10th Revision (ICD-10), was utilized to detect deaths related to cardiovascular issues, with the NCHS assigning heart diseases to codes 054–064 and cerebrovascular diseases to code 070 [13].

### Statistical analysis

R software (version 4.2.2) was used to conduct the statistical analysis. In accordance with the NHANES complex sampling design, analyses were conducted using sample weights, clustering, and stratification, as required for the correct analysis of NHANES data [14]. Normality of the CHG index was evaluated using the Shapiro–Wilk test (Supplementary Material 1, cr.elmerpub.com). Participants in the research were organized into four groups using quartiles (Q1–Q4) of the CHG index among total cohort and two age subgroups (≤ 50 years or > 50 years). Continuous variables were described using the mean and standard deviation (SD), while categorical variables were summarized as frequency and percentage. Baseline characteristics across the CHG quartile groups were compared using one-way analysis of variance (ANOVA) for continuous variables and the Pearson Chi-square test for categorical variables. The incidence rates of mortality (all-cause and cardiovascular) for each CHG quartile group were calculated over the entire follow-up period. To assess the independent predictive value of the CHG index (continuous and categorical variables), we constructed both univariate and multivariate Cox proportional hazards regression models, adjusting for potential confounding factors across three models. Model 1 did not include any adjustments. Model 2 accounted for age, race, gender, education level, and the family income-poverty ratio. Model 3 included all adjustments from model 2 and added factors such as hypertension, coronary heart disease, congestive heart failure (HF), MI, angina pectoris, stroke, smoking status, and alcohol consumption. The proportional hazards assumption for the Cox models was tested using scaled Schoenfeld residuals. No significant violations were detected (global test P > 0.05). For variables included in the Cox proportional hazards models, where the proportion of missing values was less than 10% (Supplementary Material 2, cr.elmerpub.com), multiple imputation using random forest algorithm (missForest package) was employed. Kaplan–Meier survival curves were employed to investigate the association between CHG and mortality using the log-rank test. Restricted cubic splines (RCSs) and smooth curve fitting with four knots (5%, 35%, 65%, 95%) were employed to explore the connection between the CHG index and mortality. Analyses were conducted by stratifying participants according to gender, age, race (White, Black, Mexican, or other), education level, hypertension status, and lifestyle factors like smoking and alcohol use. A model including an age × CHG interaction term was also created to evaluate the interaction between age and CHG on a continuous scale. Using the R package interaction–RCS, splines were developed based on the estimated hazard ratios (HRs) from a Cox model that incorporated a CHG–age interaction term. HRs for mortality per 1-unit increase in CHG were also calculated according to age categories (≤ 50 years and > 50 years). Two separate sensitivity analyses were carried out: one excluding patients who died within the initial 24 months of follow-up and another excluding those with a history of CVD. Statistical significance was considered as a P value of less than 0.05.

## Results

### Baseline characteristics of study participants

The baseline characteristics of the study participants (n = 14,369), categorized by CHG index quartiles and divided into age groups (≤ 50 years, n = 5,678; > 50 years, n = 8,691) were displayed (Supplementary Materials 3–5, cr.elmerpub.com). Table 1 presented the initial characteristics of the younger cohort (n = 5,678) divided into quartiles based on the CHG index. The group of participants had a median age of 38 years, with 58.1% identified as male. Male predominance was more pronounced in higher CHG index (ranging from 42.1% to 69.9%), while BMI increased progressively (ranging from 26 to 31). The racial composition differed greatly among quartiles, with the percentage of Mexican participants rising from 18.7% to 26.1%, and Black participants decreasing from 31.9% to 15.7%. Educational attainment demonstrated an inverse relationship with CHG index: higher proportions of less educated individuals were observed in the upper quartiles. CV comorbidities exhibited differing prevalence patterns across the quartile groups: HF (1.0% to 1.7%), MI (0.9% to 2.5%), angina pectoris (0.6% to 1.9%), congestive heart failure (CHD) (0.6% to 1.2%), and stroke (1.1% to 2.0%). Smoking status indicated upward trend in current smokers, increasing from 21.1% to 32.1%, while the proportion of never smokers decreased from 63.9% to 49.6% as the CHG index rose. The prevalence of hypertension elevated from 17.8% to 32.7% across quartiles. Furthermore, significant differences were observed across multiple biochemical parameters. Additionally, metabolic parameters and lipid profiles demonstrate particularly strong associations with the CHG index.

**Table 1:** Baseline Characteristics of Study Individuals Aged ≤ 50 Years According to Quartiles of the CHG Index

Characteristic	Quartiles of CHG index					P value
	Overall	Q1 (3.07, 5.12)	Q2 (5.12, 5.37)	Q3 (5.37, 5.64)	Q4 (5.64, 8.02)	
N	5,678	1,420	1,419	1,419	1,420	
Age, years	38 (29, 45)	34 (24, 43)	36 (27, 44)	39 (31, 45)	40 (33, 45)	< 0.001
Family poverty income ratio	1.83 (0.96, 3.69)	1.86 (0.95, 3.72)	1.83 (0.94, 3.74)	1.87 (1.02, 3.81)	1.77 (0.95, 3.42)	0.202
BMI, kg/m^2^	29 (25, 34)	26 (23, 31)	29 (25, 34)	30 (27, 35)	31 (28, 36)	< 0.001
Waist circumference, cm	99 (90, 111)	90 (80, 102)	98 (89, 109)	102 (94, 113)	106 (97, 118)	< 0.001
Gender, n (%)						< 0.001
Female	2,380 (41.9%)	822 (57.9%)	629 (44.3%)	501 (35.3%)	428 (30.1%)	
Male	3,298 (58.1%)	598 (42.1%)	790 (55.7%)	918 (64.7%)	992 (69.9%)	
Race, n (%)						< 0.001
Mexican American	1,321 (23.3%)	266 (18.7%)	336 (23.7%)	349 (24.6%)	370 (26.1%)	
Non-Hispanic Black	1,241 (21.9%)	453 (31.9%)	319 (22.5%)	246 (17.3%)	223 (15.7%)	
Non-Hispanic White	1,955 (34.4%)	408 (28.7%)	481 (33.9%)	519 (36.6%)	547 (38.5%)	
Other race	1,161 (20.4%)	293 (20.6%)	283 (19.9%)	305 (21.5%)	280 (19.7%)	
Education, n (%)	5,258/5,678	1,247/1,420	1,246/1,419	1,355/1,419	1,374/1,420	< 0.001
Less than 9th grade	525 (10.0%)	88 (7.1%)	113 (8.8%)	143 (10.6%)	181 (13.2%)	
9th–11th grade	895 (17.0%)	178 (14.3%)	219 (17.1%)	244 (18.0%)	254 (18.5%)	
High school graduate or equivalent	1,244 (23.7%)	270 (21.7%)	287 (22.4%)	332 (24.5%)	355 (25.8%)	
Some college or above	2,594 (49.3%)	711 (57.0%)	663 (51.7%)	636 (46.9%)	584 (42.5%)	
Miss	420	173	137	64	46	
Cerebrovascular disease, n (%)						< 0.001
Congestive heart failure	59 (1.1%)	12 (1.0%)	12 (0.9%)	11 (0.8%)	24 (1.7%)	
Coronary heart disease	41 (0.8%)	8 (0.6%)	8 (0.6%)	9 (0.7%)	16 (1.2%)	
Angina pectoris	49 (0.9%)	8 (0.6%)	8 (0.6%)	7 (0.5%)	26 (1.9%)	
Myocardial infarction	69 (1.3%)	11 (0.9%)	11 (0.9%)	12 (0.9%)	35 (2.5%)	
Stroke	63 (1.2%)	14 (1.1%)	9 (0.7%)	13 (1.0%)	27 (2.0%)	
Diabetes status, n (%)						< 0.001
Diabetes	926 (16.3%)	72 (5.1%)	110 (7.8%)	175 (12.3%)	569 (40.1%)	
Prediabetes	4,752 (83.7%)	1,348 (94.9%)	1,309 (92.2%)	1,244 (87.7%)	851 (59.9%)	
Smoking status, n (%)	5,384/5,678	1,306/1,420				< 0.001
Current	1,430 (26.6%)	275 (21.1%)	352 (26.6%)	360 (26.2%)	443 (32.1%)	
Former	914 (17.0%)	196 (15.0%)	208 (15.7%)	257 (18.7%)	253 (18.3%)	
Never	3,040 (56.5%)	835 (63.9%)	763 (57.7%)	756 (55.1%)	686 (49.6%)	
Miss	294	114	96	46	38	
Hypertension, n (%)						< 0.001
No	4,298 (76.0%)	1,162 (82.2%)	1,097 (77.5%)	1,085 (77.1%)	954 (67.3%)	
Yes	1,357 (24.0%)	252 (17.8%)	319 (22.5%)	323 (22.9%)	463 (32.7%)	
Miss	23	6	3	11	3	
Alcohol consumption, n (%)						< 0.001
Heavy	319 (6.0%)	85 (6.6%)	97 (7.4%)	70 (5.2%)	67 (4.9%)	
Moderate	2,102 (39.6%)	382 (29.5%)	499 (38.3%)	593 (44.1%)	628 (46.1%)	
Mild	2,276 (42.9%)	665 (51.3%)	556 (42.7%)	525 (39.0%)	530 (38.9%)	
Never	609 (11.5%)	164 (12.7%)	151 (11.6%)	157 (11.7%)	137 (10.1%)	
Miss	372	124	116	74	58	
Laboratory measurements						
TC, mg/dL	190 (165, 219)	166 (145, 189)	183 (162, 205)	197 (177, 223)	218 (192, 245)	< 0.001
TG, mmol/L	1.25 (0.85, 1.92)	0.75 (0.58, 1.03)	1.08 (0.82, 1.47)	1.45 (1.11, 1.94)	2.21 (1.58, 3.29)	< 0.001
HDLC, mmol/L	1.22 (1.01, 1.45)	1.58 (1.37, 1.81)	1.29 (1.14, 1.45)	1.11 (1.01, 1.24)	0.96 (0.84, 1.09)	< 0.001
LDLC, mmol/L	2.97 (2.38, 3.60)	2.30 (1.91, 2.73)	2.92 (2.46, 3.36)	3.28 (2.77, 3.83)	3.57 (2.97, 4.19)	< 0.001
HbA1C, %	5.60 (5.30, 5.80)	5.40 (5.20, 5.70)	5.50 (5.20, 5.70)	5.50 (5.30, 5.80)	5.80 (5.40, 7.00)	< 0.001
Fasting glucose, mg/dL	104 (101, 112)	101 (97, 105)	103 (100, 108)	105 (101, 111)	114 (105, 156)	< 0.001
Insulin, pmol/L	13 (8, 21)	9 (6, 14)	12 (7, 19)	14 (9, 22)	18 (12, 28)	< 0.001
TBil, umol/L	10.3 (8.6, 13.7)	10.3 (8.6, 13.7)	10.3 (8.6, 13.7)	10.3 (8.6, 13.7)	10.3 (8.6, 13.7)	0.024
LDH, mmol/L	127 (112, 145)	124 (110, 142)	126 (111, 145)	128 (113, 146)	129 (114, 147)	< 0.001
BUN, mmol/L	4.28 (3.21, 5.00)	3.93 (3.21, 5.00)	3.93 (3.21, 5.00)	4.28 (3.57, 5.00)	4.28 (3.57, 5.36)	< 0.001
UA, µmol/L	327 (274, 387)	286 (244, 339)	327 (274, 381)	345 (292, 405)	351 (296, 410)	< 0.001
SCr, µmol/L	72 (62, 84)	71 (59, 81)	72 (62, 85)	73 (63, 86)	72 (62, 86)	0.006
Serum iron, µmol/L	15 (11, 19)	14 (10, 19)	15 (11, 19)	15 (11, 19)	15 (11, 19)	0.401
Serum potassium, mmol/L	4.00 (3.80, 4.20)	3.98 (3.80, 4.20)	4.00 (3.80, 4.20)	4.00 (3.80, 4.20)	4.00 (3.80, 4.20)	< 0.001
GGT, IU/L	23 (16, 35)	17 (12, 24)	21 (15, 30)	25 (18, 37)	31 (22, 48)	< 0.001
AST, IU/L	23 (19, 28)	21 (18, 26)	22 (19, 27)	23 (19, 29)	25 (20, 31)	< 0.001
ALT, IU/L	23 (17, 34)	18 (14, 24)	22 (17, 31)	26 (19, 37)	30 (21, 44)	< 0.001
CRP, mmol/L	0.42 (0.14, 1.45)	0.31 (0.08, 1.13)	0.42 (0.12, 1.51)	0.46 (0.15, 1.42)	0.51 (0.21, 1.62)	0.177

ALT: alanine aminotransferase; AST: aspartate aminotransferase; BUN: blood urea nitrogen; CHG: cholesterol, high-density lipoprotein, and glucose; HDL: high-density lipoprotein cholesterol; LDH: lactate dehydrogenase; LDL-C: low-density lipoprotein cholesterol; HbA1C: hemoglobin A1c; TBil: total bilirubin; TC: total cholesterol; UA: uric acid; GGT: γ-glutamyl transferase; CRP: C-reactive protein; SCr: serum creatinine.

### Mortality outcomes distributed across CHG quartiles

**Figure 2:**
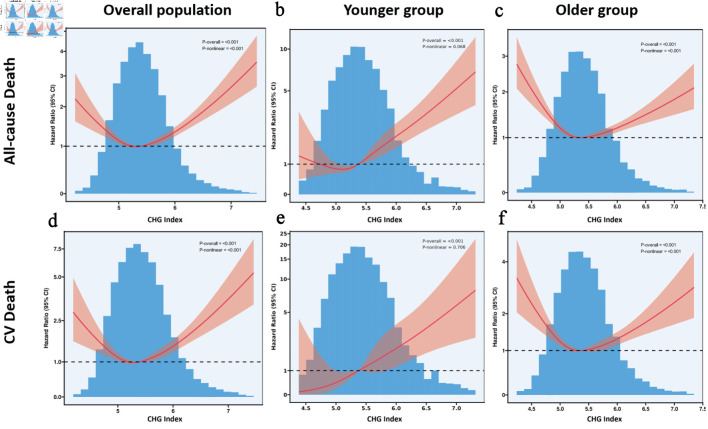
(a–f) Restricted cubic spline models analyzed the relationship between cholesterol, high-density lipoprotein, glucose index, and mortality. CHG: cholesterol, high-density lipoprotein, and glucose; CI: confidence interval; CV: cardiovascular.

As CHG quartiles increase, there is a progressive rise in both all-cause and cardiovascular mortality, with all P values under 0.001 (Supplementary Material 6, cr.elmerpub.com). Notably, Q4 of CHG shows significantly higher rates of all-cause mortality and cardiovascular mortality, at 23.4% and 8.0%, respectively. Both the younger and older groups showed a similar trend. In the total population, Cox proportional hazards regression models with RCSs and smooth curve fitting revealed U-shaped associations between CHG index and both all-cause mortality (Fig. 2a) and cardiovascular (CV) mortality (Fig. 2d). In a separate analysis of populations with diabetes and prediabetes (Supplementary Material 7, cr.elmerpub.com), we also observed that the U-shaped relationship between the CHG index and both all-cause and CV mortality persisted (both P for non-linear < 0.001). Among participants enrolled in the study, there was a significant interaction between CHG and age (CHG × age interaction P < 0.001), such that patients with lower age had higher hazards of death with higher CHG index (Supplementary Materials 8G, H, 9, 10, cr.elmerpub.com). Accounting for the effects of age, additional RCS analyses indicated a significant positive link between CHG and all mortality outcomes for those 50 years old or younger, without any non-linear relationship detected (Fig. 2b, e). The U-shaped relationship was still present in those > 50 years of age (Fig. 2c, f).

### Associations between CHG and mortality in diabetes and prediabetes patients

Among all individuals, the CHG index was associated with greater risk of mortality in all age groups but had a significantly stronger association in the younger group. For patients with age ≤ 50 years, Table 2 shows that higher CHG index (per 1-unit) was associated with greater risk of all-cause mortality (HR = 2.46; 95% confidence interval (CI), 1.94–3.12; P < 0.001) and CV mortality (HR = 3.26; 95% CI, 2.11–5.04; P < 0.001). The quartiles of the CHG index (Q4) were significantly associated with an increased risk of all-cause, and cardiovascular mortality compared to Q1 of CHG (all-cause mortality: HR = 2.66; 95 % CI, 1.78–3.96; CV mortality: HR = 4.77; 95 % CI, 1.84–12.3). The association between higher CHG and increased mortality risk was further supported by models 2 and 3. However, the multivariable Cox proportional hazards regression models validated the absence of a direct connection between CHG and mortality among the older individuals. The Cox regression model results and subgroup analysis regarding the link between CHG and mortality in the study population aged 18–85 years are presented here (Supplementary Materials 9–11, cr.elmerpub.com). The Kaplan–Meier survival curves revealed that patients in the Q4 of the CHG index had a significantly higher risk of mortality across all age groups (Supplementary Materials 8C, D, 12, cr.elmerpub.com).

**Table 2:** The Association of the CHG Index With Mortality in Individuals With Diabetes or Prediabetes ≤ 50 Years and > 50 Years

Characteristic	Model 1			Model 2			Model 3		
	HR	95% CI	P value	HR	95% CI	P value	HR	95% CI	P value
All–cause mortality									
Diabetes or prediabetes with 18–50 years									
CHG index (continuous)	2.46	1.94–3.12	< 0.001	2.06	1.57–2.69	< 0.001	1.86	1.40–2.47	< 0.001
CHG index									
Q1	1 (reference)			1 (reference)			1 (reference)		
Q2	1.23	0.77–1.95	0.383	1.05	0.64–1.72	0.841	0.96	0.58–1.59	0.885
Q3	1.24	0.79–1.95	0.359	1.03	0.63–1.66	0.920	0.95	0.58–1.56	0.840
Q4	2.66	1.78–3.96	< 0.001	2.02	1.30–3.12	0.002	1.68	1.06–2.65	0.026
P for trend			< 0.001			< 0.001			0.005
Diabetes or prediabetes with 51–85 years									
CHG index (continuous)	1.00	0.92–1.10	0.929	1.21	1.09–1.33	< 0.001	1.21	1.09–1.35	< 0.001
CHG index									
Q1	1 (reference)			1 (reference)			1 (reference)		
Q2	0.80	0.71–0.90	< 0.001	0.84	0.74–0.94	0.004	0.84	0.74–0.95	0.005
Q3	0.77	0.68–0.86	< 0.001	0.81	0.72–0.92	< 0.001	0.82	0.72–0.93	0.002
Q4	0.92	0.83–1.03	0.149	1.09	0.97–1.23	0.134	1.10	0.97–1.24	0.144
P for trend			0.209			0.139			0.125
CV mortality									
Diabetes or prediabetes with 18–50 years									
CHG index (continuous)	3.26	2.11–5.04	< 0.001	2.81	1.76–4.49	< 0.001	2.54	1.55–4.15	< 0.001
CHG index									
Q1	1 (reference)			1 (reference)			1 (reference)		
Q2	1.72	0.58–5.13	0.332	1.48	0.49–4.45	0.485	1.23	0.40–3.81	0.723
Q3	2.10	0.74–5.96	0.164	1.62	0.56–4.69	0.372	1.46	0.50–4.29	0.494
Q4	4.77	1.84–12.3	0.001	3.45	1.29–9.22	0.013	2.69	0.98–7.36	0.054
P for trend			< 0.001			0.003			0.015
Diabetes or prediabetes with 51–85 years									
CHG index (continuous)	1.09	0.93–1.27	0.281	1.41	1.19–1.67	< 0.001	1.32	1.10–1.59	0.003
CHG index									
Q1	1 (reference)			1 (reference)			1 (reference)		
Q2	0.78	0.64, 0.95	0.013	0.80	0.65, 0.99	0.036	0.78	0.63, 0.97	0.028
Q3	0.76	0.63, 0.93	0.006	0.83	0.67, 1.02	0.074	0.79	0.63, 0.98	0.031
Q4	0.96	0.79, 1.15	0.634	1.20	0.98, 1.47	0.072	1.14	0.92, 1.41	0.221
P for trend			0.795			0.053			0.180

Model 1: no covariates were adjusted. Model 2 accounted for age, race, gender, education level, and family income-poverty ratio. Model 3: model 2 + hypertension, CVD (coronary heart disease, congestive heart failure, myocardial infarction, angina pectoris, stroke), smoking status, and alcohol consumption. HR: hazard ratio; CI: confidence interval; CHG: cholesterol, high-density lipoprotein, and glucose; CV: cardiovascular.

### Sensitivity analyses

In sensitivity analyses, the significant association between CHG and mortality outcomes persisted even after excluding participants who died within the first 24 months of follow-up and those with a history of CVD at baseline (Supplementary Materials 13, 14, cr.elmerpub.com).

### Subgroup analysis of CHG in predicting mortality

Tables 3 and 4 present subgroup analyses evaluating the association between CHG and the risk of all-cause mortality and CV mortality, respectively, in younger individuals. The interactions between CHG and various covariates—including gender, age, race, education level, history of cerebrovascular disease, smoking status, hypertension, and alcohol consumption—were not statistically significant (P > 0.05 for interaction). Similarly, the relationship between CHG quartiles and mortality risk was not significantly modified by these factors (P > 0.05 for interaction; Supplementary Materials 15, 16, cr.elmerpub.com).

**Table 3:** Subgroup Analysis of CHG Index in Predicting All-Cause Mortality in Individuals With Diabetes or Prediabetes ≤ 50 Years

Subgroup	N	HR (95% CI)	P value	P for interaction
Overall	5,678	2.46 (1.94–3.12)	< 0.001	
Age (years)				0.054
< 35	2,283	3.54 (2.05–6.09)	< 0.001	
≥ 35	3,395	1.94 (1.46–2.56)	< 0.001	
Gender				0.641
Female	2,380	2.66 (1.77–4.00)	< 0.001	
Male	3,298	2.34 (1.73–3.16)	< 0.001	
Race				0.75
Mexican American	1,321	2.89 (1.50–5.58)	0.001	
Non-Hispanic Black	1,241	2.17 (1.42–3.32)	< 0.001	
Non-Hispanic White	1,955	2.90 (2.03–4.15)	< 0.001	
Other race	1,161	2.25 (1.01–4.99)	0.047	
Education level				0.2
Less than 9th grade	525	1.26 (0.52–3.09)	0.611	
9th–11th grade	895	1.96 (1.21–3.18)	0.006	
High school graduate or equivalent	1,244	3.24 (1.97–5.32)	< 0.001	
Some college or above	2,594	2.64 (1.75–3.97)	< 0.001	
Cerebrovascular disease				0.051
No	5,482	2.50 (1.93–3.24)	< 0.001	
Yes	196	1.23 (0.63–2.42)	0.543	
Smoking status				0.206
Current	1,430	2.01 (1.41–2.87)	< 0.001	
Former	914	1.75 (0.93–3.31)	0.082	
Never	3,040	3.15 (2.04–4.84)	< 0.001	
Hypertension				0.3
No	4,298	2.49 (1.79–3.45)	< 0.001	
Yes	1,357	1.91 (1.31–2.77)	0.001	
Alcohol consumption				0.283
Heavy	319	0.88 (0.08–10.12)	0.92	
Moderate	2,102	1.98 (1.30–3.03)	0.001	
Mild	2,276	2.67 (1.88–3.80)	< 0.001	
Never	609	3.93 (2.12–7.28)	< 0.001	

CHG: cholesterol, high-density lipoprotein, and glucose; HR: hazard ratio; CI: confidence interval.

**Table 4:** Subgroup Analysis of CHG Index in Predicting Cardiovascular Mortality in Individuals With Diabetes or Prediabetes ≤ 50 Years

Subgroup	N	HR (95% CI)	P value	P for interaction
Overall	5,678	3.26 (2.11–5.04)	< 0.001	
Age (years)				0.037
< 35	2,283	6.57 (2.93–14.71)	< 0.001	
≥ 35	3,395	2.33 (1.36–3.99)	0.002	
Gender				0.927
Female	2,380	3.02 (1.08–8.42)	0.035	
Male	3,298	3.10 (1.89–5.10)	< 0.001	
Race				0.403
Mexican American	1,321	6.05 (2.14–17.06)	0.001	
Non-Hispanic Black	1,241	2.33 (1.14–4.76)	0.02	
Non-Hispanic White	1,955	4.13 (2.00–8.55)	< 0.001	
Other race	1,161	5.12 (1.09–24.09)	0.039	
Education level				0.229
Less than 9th grade	525	1.76 (0.18–17.05)	0.624	
9th–11th grade	895	1.68 (0.67–4.22)	0.27	
High school graduate or equivalent	1,244	5.26 (2.21–12.50)	< 0.001	
Some college or above	2,594	4.05 (2.08–7.88)	< 0.001	
Cerebrovascular disease				0.082
No	5,482	3.53 (2.19–5.69)	< 0.001	
Yes	196	1.23 (0.40–3.73)	0.718	
Smoking status				0.249
Current	1,430	2.36 (1.16–4.83)	0.018	
Former	914	1.93 (0.54–6.89)	0.309	
Never	3,040	4.76 (2.47–9.14)	< 0.001	
Hypertension				0.096
No	4,298	4.14 (2.34–7.34)	< 0.001	
Yes	1,357	1.91 (0.94–3.88)	0.072	
Alcohol consumption				0.725
Moderate/heavy	2,421	2.58 (1.23–5.44)	0.013	
Mild	2,276	3.66 (1.84–7.27)	< 0.001	
Never	609	3.77 (1.44–9.81)	0.007	

CHG: cholesterol, high-density lipoprotein, and glucose; HR: hazard ratio; CI: confidence interval.

## Discussion

In this study, we initially demonstrated the association between the CHG index and both all-cause and cardiovascular mortality, showing a U-shaped relationship in those with diabetes or prediabetes. There was a significant association between a higher CHG index and increased mortality among those aged 50 years or below. The findings of this study highlight the critical role of the CHG index in identifying younger diabetic or prediabetic patients who are at increased risk of mortality, thus supporting further assessments and more proactive treatment measures.

Early-onset diabetes mellitus has an earlier risk of complications and a higher risk of early death [15, 16]. The Framingham study found that as the onset of diabetes occurs at younger ages, the mortality rates of CVDs and non-CVDs increase accordingly [17]. Patients with early-onset T2DM experience quicker β-cell dysfunction, faster development of diabetic complications, and a greater occurrence of obesity and related health issues compared to those with late-onset T2DM. Patients diagnosed with early-onset diabetes exhibit an elevated risk for complications affecting both small and large blood vessels [18]. Studies show that the CHG index predicts short-term stroke mortality and long-term CVD risk and outperforms TyG and lipid accumulation product (LAP) for diabetic retinopathy [11, 19, 20]. This suggests that the CHG index is a promising biomarker for systemic metabolic dysregulation, with links to cardiovascular and metabolic disease outcomes like stroke, aortic stenosis, and metabolic syndrome [21–23]. Our results might broaden our comprehension of the link between the CHG index and mortality risk in people with early-onset diabetes, offering new perspectives for future research. Similarly, a study showed that CHG index were positively correlated with metabolic dysfunction-associated steatotic liver disease (MASLD) in young people [24]. This suggests that our findings are intentional: young people may be more susceptible to metabolic disorders due to their active metabolic processes. Changes in the CHG index can significantly affect their health, as they typically have fewer chronic diseases. Specifically, an increase of one unit in the baseline CHG index was associated with an 86% higher risk of death from all causes and a 154% higher risk of cardiovascular death, underscoring the uniqueness and importance of our findings.

Currently, the precise biological mechanisms by which the CHG index predicts the risk of CVD remain to be fully elucidated. In our study, we constructed comprehensive evaluation models, including Cox regression and RCS models, to further explore the association between the CHG index and CVD risk. It is widely recognized that cholesterol levels substantially influence CVD risk [25]. Higher TC and LDL levels are linked to a higher mortality rate from CVD, whereas HDL shows a negative correlation with CVD risk [26, 27]. Furthermore, our research found a positive association between the CHG index and BMI, HbA1c, TG, and LDL-C. These findings imply that the link between the CHG index and adverse clinical outcomes may be mediated by established traditional CVD risk factors.

The CHG index, which includes TC, FBG, and HDL-C, might offer benefits in evaluating IR and metabolic disorders by integrating these additional lipid parameters. Furthermore, IR is a prevalent metabolic disorder in obese patients and plays a significant role in progression of dyslipidemia [27]. IR contributes to CVD development in both the general population and early-onset diabetes patients, and it also predicts CV prognosis in individuals with CVD [28–31]. The raised lipid and glucose levels are considered significant risk factors for CVD, and they are known to be associated with inflammation [32–34]. Elevated TyG index levels have been found to be associated with inflammatory and oxidative conditions, which might shed light on the greater risks of T2DM and CVD, according to one study [35]. The CHG index may have a notable benefit over the TyG index due to its incorporation of a wider array of lipid parameters, particularly TC and HDL-C. However, there is a current shortage of studies investigating how the CHG index relates to inflammation.

In alignment with previous research [29, 36], our study corroborated a U-shaped relationship between the CHG index and mortality from all causes and cardiovascular issues in people with diabetes and prediabetes. This finding suggests that patients face higher mortality risks with both very high and very low CHG indices, while maintaining a balanced CHG index is vital. There are likely multiple mechanisms for this finding. Abnormal blood glucose levels can adversely affect the prognosis of patients with diabetes. Elevated FBG levels have been linked to an increased long-term absolute risk of CVD, and lower FBG levels might be associated with a higher occurrence of negative outcomes [37–39]. HDL-C also has antioxidant and anti-inflammatory effects and improves vascular endothelial function [40]. Low HDL-C levels may weaken its protective effect on the cardiovascular system [41]. Meanwhile, studies on large populations have shown that individuals carrying one copy of the P376L variant have notably higher plasma HDL-C levels, potentially increasing the risk of atherosclerosis [42]. Extremely low levels of CHG index (high HDL cholesterol and low fasting glucose) may suggest inadequate nutritional status and have been linked to increased long-term mortality from all causes [43]. These possibilities remain unclear, and causal relationships are unestablished, making the mechanisms underlying the U-shaped curve hypothetical.

### Limitations

This study is subject to several limitations. Firstly, it relies on data from the NHANES database, which, while representative, may not fully encompass the CVD risk factors and population characteristics found in other countries or regions. Secondly, the outcomes of the study were based on self-reported questionnaires, which may introduce bias in the data collection process. Thirdly, as an observational study, it cannot definitively establish causality. Despite efforts to control for confounding variables through multivariate adjustments and subgroup analyses, residual confounding factors—such as lipid-lowering drugs, diabetic therapy, duration of therapy, and compliance, for which information is lacking—may still influence patient prognosis. Finally, this analysis focuses exclusively on the prognostic value of the baseline CHG index, leaving the question of whether changes in the CHG index during follow-up can also predict mortality unanswered, necessitating further investigation.

### Conclusions

An elevated CHG index represents a significant prognostic marker for predicting the risk of all-cause and CV mortality among young individuals with diabetes or prediabetes. Further research is warranted to investigate whether targeted interventions designed to modify the CHG index may lead to improved clinical outcomes in this patient population.

## Supplementary Material

Suppl 1 Density plot of CHG index by age group.

Suppl 2 Proportions of missing value.

Suppl 3 Baseline characteristics according to the CHG index quartiles in total cohorts (aged 18 to 85 years).

Suppl 4 Baseline levels of laboratory characteristics according to the CHG index quartiles in total cohorts (aged 18 to 85 years).

Suppl 5 Baseline characteristics grouped by age group.

Suppl 6 Distribution of mortality outcomes by CHG quartiles in diabetes and prediabetes patients.

Suppl 7 Association between CHG index and all-cause mortality (A) and CV mortality (B) in diabetes and all-cause mortality (C) and CV mortality (D) in pre-diabetes population.

Suppl 8 Association of CHG index with all-cause mortality and cardiovascular mortality among diabetes or prediabetes population.

Suppl 9 Subgroup analysis of exploring the interaction between CHG index and all-cause mortality outcomes in total cohorts (aged 18 to 85 years).

Suppl 10 Subgroup analysis of exploring the interaction between CHG index and CV-related mortality outcomes in total cohorts (aged 18 to 85 years).

Suppl 11 HRs (95% CIs) for mortality according to the CHG index quartiles in total cohorts (aged 18 to 85 years).

Suppl 12 Kaplan–Meier survival curve analysis for the all-cause mortality and CV mortality.

Suppl 13 Sensitivity analysis of CHG index and mortality outcomes in patients after excluding patients died within the first 24-month follow-up.

Suppl 14 Sensitivity analysis of CHG index and mortality outcomes in patients after excluding patients after excluding patients with CVD.

Suppl 15 Subgroup analysis of exploring the interaction between CHG index (quartile 4) and all-cause mortality in population aged 18 to 50 years.

Suppl 16 Subgroup analysis of exploring the interaction between CHG index (quartile 4) and CV mortality in population aged 18 to 50 years.

## Data Availability

This publication includes the data and original contributions that support the study’s findings. Additional information can be obtained from the corresponding authors upon reasonable request.

## Abbreviations

ALB: albumin
ALT: alanine aminotransferase
AST: aspartate aminotransferase
AUC: area under the curve
BUN: blood urea nitrogen
CHG: cholesterol, high-density lipoprotein, and glucose
CVD: cardiovascular disease
eGFR: estimated glomerular filtration rate
FBG: fasting blood glucose
HDL: high-density lipoprotein cholesterol
HOMA-IR: homeostatic model assessment for insulin resistance
ICD-10: the International Classification of Diseases, 10th Revision
IR: insulin resistance
LDH: lactate dehydrogenase
LDL-C: low-density lipoprotein cholesterol
NHANES: National Health And Nutrition Examination Survey
PIR: poverty-to-income ratio
RCS: restricted cubic spline
TBil: total bilirubin
TC: total cholesterol
TyG: triglyceride-glucose
UA: uric acid
