# Supplementary figures and images for: The Association Between Cholesterol, High-Density Lipoprotein, and Glucose Index and Mortality in Young and Middle-Aged Adults With Diabetes or Prediabetes: NHANES Data (1999–2018)
Source: Cardiol Res. 2026 Apr 15;17(2):136–48. doi: 10.14740/cr2190 (PMC13094157; doi:10.14740/cr2190)

**Suppl 1.** Density plot of CHG index by Age group


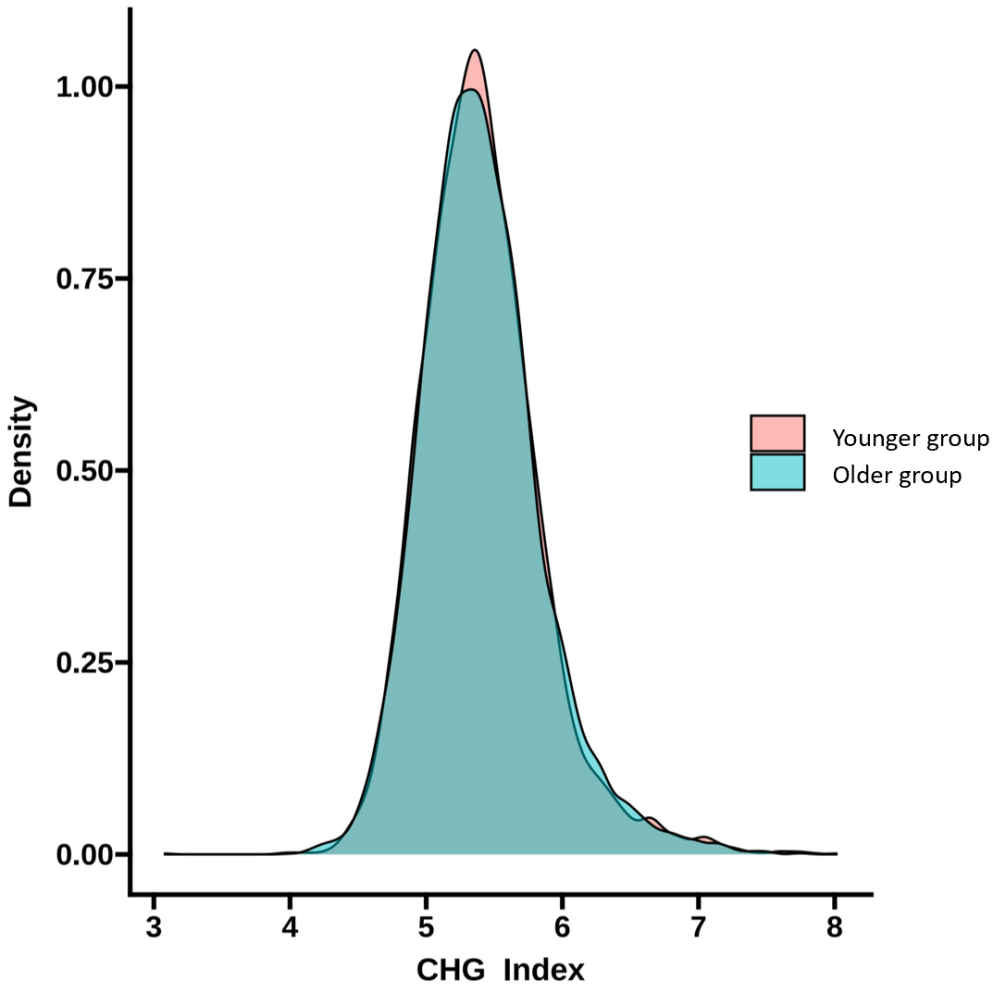

Supplement: Suppl 1 — Density plot of CHG index by age group. [file cr-17-02-136-s001.docx]

**Suppl 12.** Kaplan–Meier survival curve analysis for the all-cause mortality and CV mortality.


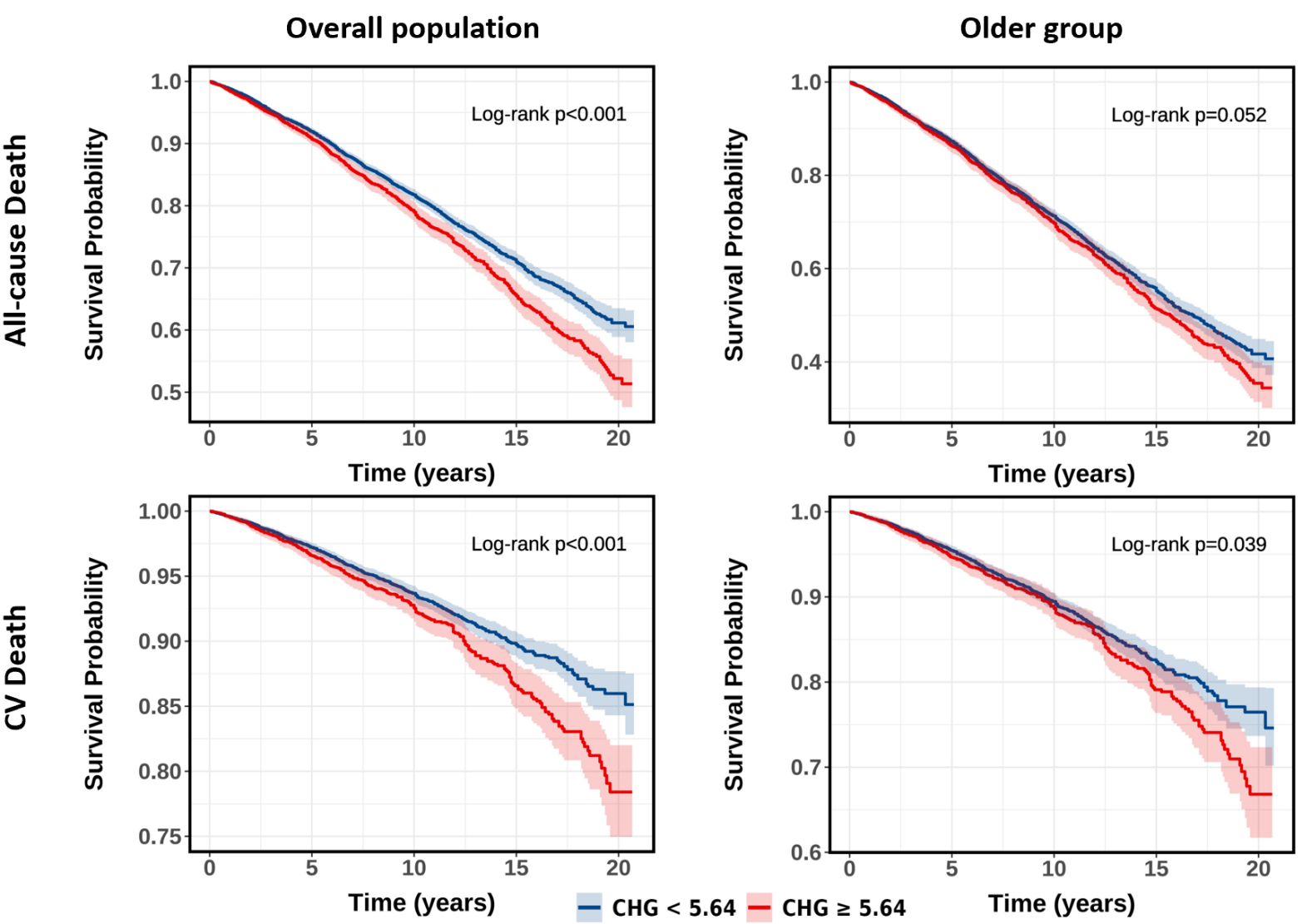

Supplement: Suppl 12 — Kaplan–Meier survival curve analysis for the all-cause mortality and CV mortality. [file cr-17-02-136-s012.docx]
