# Supplementary material for: The Association Between Cholesterol, High-Density Lipoprotein, and Glucose Index and Mortality in Young and Middle-Aged Adults With Diabetes or Prediabetes: NHANES Data (1999–2018)
Source: Cardiol Res. 2026 Apr 15;17(2):136–48. doi: 10.14740/cr2190 (PMC13094157; doi:10.14740/cr2190)
Supplement: Suppl 2 — Proportions of missing value. [file cr-17-02-136-s002.docx]

**Suppl 2.** Proportions of missing value

| Characteristics | Overall participants (n = 14369) | |
| --- | --- | --- |
|  | N | Percentage, % |
| Poverty income ratio | 1352/14369 | 9.4% |
| Education level | 441/14369 | 3.1% |
| Smoking status | 306/14369 | 2.1% |
| Hypertension | 26/14369 | 0.2% |
| Alcohol consumption | 765/14369 | 5.3% |
