# Supplementary material for: The Association Between Cholesterol, High-Density Lipoprotein, and Glucose Index and Mortality in Young and Middle-Aged Adults With Diabetes or Prediabetes: NHANES Data (1999–2018)
Source: Cardiol Res. 2026 Apr 15;17(2):136–48. doi: 10.14740/cr2190 (PMC13094157; doi:10.14740/cr2190)
Supplement: Suppl 3 — Baseline characteristics according to the CHG index quartiles in total cohorts (aged 18 to 85 years). [file cr-17-02-136-s003.docx]

**Suppl 3.** Baseline characteristics according to the CHG index quartiles in total cohorts (aged 18 to 85 years)

| **Characteristic** | **Quartiles of CHG index** | | | | | **p-value** |
| --- | --- | --- | --- | --- | --- | --- |
|  | **Overall** | **Q1(3.07, 5.12)** | **Q2(5.12, 5.37)** | **Q3(5.37, 5.65)** | **Q4(5.65, 8.02)** |  |
| **N(%)** | 14,369 | 3,591 | 3,593 | 3,592 | 3,593 |  |
| **Age, years** | 56 (42, 68) | 58 (39, 71) | 57 (41, 69) | 55 (42, 68) | 56 (44, 66) | 0.368 |
| **BMI, kg/m^2^** | 29 (25, 34) | 27 (23, 31) | 29 (25, 33) | 30 (27, 34) | 30 (27, 35) | <0.001 |
| **Gender, n(%)** |  |  |  |  |  | <0.001 |
| Male | 7,772 (54.1%) | 1,479 (41.2%) | 1,854 (51.6%) | 2,143 (59.7%) | 2,296 (63.9%) |  |
| Female | 6,597 (45.9%) | 2,112 (58.8%) | 1,739 (48.4%) | 1,449 (40.3%) | 1,297 (36.1%) |  |
| **Race, n(%)** |  |  |  |  |  | <0.001 |
| Mexican | 2,702 (18.8%) | 482 (13.4%) | 640 (17.8%) | 715 (19.9%) | 865 (24.1%) |  |
| Black | 2,997 (20.9%) | 1,038 (28.9%) | 744 (20.7%) | 641 (17.8%) | 574 (16.0%) |  |
| White | 6,019 (41.9%) | 1,429 (39.8%) | 1,518 (42.2%) | 1,565 (43.6%) | 1,507 (41.9%) |  |
| Other | 2,651 (18.4%) | 642 (17.9%) | 691 (19.2%) | 671 (18.7%) | 647 (18.0%) |  |
| **Education, n(%)** |  |  |  |  |  | <0.001 |
| Less than high school | 4,269 (30.7%) | 883 (25.9%) | 974 (28.2%) | 1,100 (31.2%) | 1,312 (37.1%) |  |
| High school graduate or equivalent | 3,290 (23.6%) | 778 (22.8%) | 813 (23.6%) | 855 (24.3%) | 844 (23.8%) |  |
| Some college or above | 6,369 (45.7%) | 1,754 (51.4%) | 1,662 (48.2%) | 1,570 (44.5%) | 1,383 (39.1%) |  |
| **Alcohol, n(%)** |  |  |  |  |  | <0.001 |
| Never | 1,758 (14.3%) | 444 (14.9%) | 423 (13.9%) | 460 (14.8%) | 431 (13.5%) |  |
| Mild | 6,077 (49.4%) | 1,666 (55.9%) | 1,536 (50.6%) | 1,404 (45.2%) | 1,471 (46.1%) |  |
| Moderate/Heavy | 4,477 (36.4%) | 871 (29.2%) | 1,075 (35.4%) | 1,244 (40.0%) | 1,287 (40.4%) |  |
| **Smoking status, n(%)** |  |  |  |  |  | <0.001 |
| Never | 7,178 (51.0%) | 1,947 (56.0%) | 1,861 (53.3%) | 1,742 (49.2%) | 1,628 (45.8%) |  |
| Former | 4,084 (29.0%) | 927 (26.7%) | 983 (28.1%) | 1,058 (29.9%) | 1,116 (31.4%) |  |
| Current | 2,801 (19.9%) | 600 (17.3%) | 650 (18.6%) | 743 (21.0%) | 808 (22.7%) |  |
| **Family poverty income ratio, n(%)** | 2.00 (1.08, 3.87) | 2.12 (1.14, 4.06) | 2.11 (1.13, 4.07) | 2.10 (1.12, 4.03) | 1.80 (1.02, 3.36) | <0.001 |
| **Hypertension, n(%)** |  |  |  |  |  | <0.001 |
| No | 7,885 (55.0%) | 2,042 (57.0%) | 2,023 (56.4%) | 1,997 (55.8%) | 1,823 (50.8%) |  |
| Yes | 6,458 (45.0%) | 1,543 (43.0%) | 1,565 (43.6%) | 1,584 (44.2%) | 1,766 (49.2%) |  |
| **Diabete subtype, n(%)** |  |  |  |  |  | <0.001 |
| DM | 4,189 (29.2%) | 549 (15.3%) | 689 (19.2%) | 927 (25.8%) | 2,024 (56.3%) |  |
| Pre-DM | 10,180 (70.8%) | 3,042 (84.7%) | 2,904 (80.8%) | 2,665 (74.2%) | 1,569 (43.7%) |  |
| **Cerebro-cardiovascular disease** | | | | | | |
| **Congestive Heart Failure, n(%)** |  |  |  |  |  |  |
| No | 13,225 (95.2%) | 3,233 (94.9%) | 3,328 (96.7%) | 3,349 (95.4%) | 3,315 (93.9%) | <0.001 |
| Yes | 667 (4.8%) | 174 (5.1%) | 115 (3.3%) | 162 (4.6%) | 216 (6.1%) |  |
| **Coronary Heart Disease, n(%)** |  |  |  |  |  |  |
| No | 13,058 (94.2%) | 3,187 (93.9%) | 3,260 (94.8%) | 3,326 (94.8%) | 3,285 (93.2%) | 0.012 |
| Yes | 805 (5.8%) | 206 (6.1%) | 180 (5.2%) | 181 (5.2%) | 238 (6.8%) |  |
| **Stroke, n(%)** |  |  |  |  |  |  |
| No | 13,228 (94.9%) | 3,209 (94.1%) | 3,319 (96.1%) | 3,359 (95.3%) | 3,341 (94.3%) | 0.001 |
| Yes | 705 (5.1%) | 203 (5.9%) | 133 (3.9%) | 167 (4.7%) | 202 (5.7%) |  |
| **Myocardial infarction, n(%)** |  |  |  |  |  |  |
| No | 13,045 (93.7%) | 3,185 (93.3%) | 3,265 (94.7%) | 3,316 (94.2%) | 3,279 (92.5%) | <0.001 |
| Yes | 882 (6.3%) | 227 (6.7%) | 183 (5.3%) | 206 (5.8%) | 266 (7.5%) |  |
| **Angina pectoris, n(%)** |  |  |  |  |  |  |
| No | 13,331 (96.0%) | 3,277 (96.4%) | 3,320 (96.4%) | 3,381 (96.2%) | 3,353 (95.1%) | 0.013 |
| Yes | 550 (4.0%) | 122 (3.6%) | 123 (3.6%) | 133 (3.8%) | 172 (4.9%) |  |
| Date are presented as IQR (median) or n (%); | | | | | | |
