# Supplementary material for: The Association Between Cholesterol, High-Density Lipoprotein, and Glucose Index and Mortality in Young and Middle-Aged Adults With Diabetes or Prediabetes: NHANES Data (1999–2018)
Source: Cardiol Res. 2026 Apr 15;17(2):136–48. doi: 10.14740/cr2190 (PMC13094157; doi:10.14740/cr2190)
Supplement: Suppl 4 — Baseline levels of laboratory characteristics according to the CHG index quartiles in total cohorts (aged 18 to 85 years). [file cr-17-02-136-s004.docx]

**Suppl 4.** Baseline levels of laboratory characteristics according to the CHG index quartiles in total cohorts (aged 18 to 85 years)

| **Characteristic** | **Quartiles of CHG index** | | | | **p-value** |
| --- | --- | --- | --- | --- | --- |
|  | **Q1(3.07, 5.12)** | **Q2(5.12, 5.37)** | **Q3(5.37, 5.65)** | **Q4(5.65, 8.02)** |  |
| **HbA1C,%** | 5.70 (5.30, 5.90) | 5.70 (5.40, 6.00) | 5.70 (5.40, 6.10) | 6.20 (5.60, 7.70) | <0.001 |
| **FPG, mg/dl** | 102 (97, 107) | 105 (101, 113) | 108 (102, 119) | 128 (109, 180) | <0.001 |
| **FIN, pmol/L** | 8 (6, 13) | 11 (7, 17) | 13 (9, 20) | 16 (10, 26) | <0.001 |
| **TC, mg/dl** | 172 (148, 199) | 186 (163, 212) | 198 (174, 224) | 214 (186, 245) | <0.001 |
| **TG, mmol/L** | 0.86 (0.64, 1.14) | 1.19 (0.89, 1.57) | 1.51 (1.14, 2.00) | 2.16 (1.56, 3.11) | <0.001 |
| **HDL, mmol/L** | 1.66 (1.42, 1.94) | 1.34 (1.19, 1.55) | 1.16 (1.03, 1.32) | 1.01 (0.88, 1.16) | <0.001 |
| **LDL, mmol/L** | 2.35 (1.89, 2.82) | 2.90 (2.35, 3.41) | 3.21 (2.66, 3.78) | 3.47 (2.79, 4.16) | <0.001 |
| **ALT, IU/L** | 19 (15, 24) | 21 (16, 28) | 23 (17, 31) | 25 (19, 35) | <0.001 |
| **AST, IU/L** | 22 (19, 27) | 22 (19, 27) | 23 (19, 28) | 23 (19, 29) | 0.028 |
| **BUN, mmol/L** | 4.64 (3.57, 6.07) | 4.64 (3.90, 6.07) | 4.64 (3.93, 6.07) | 5.00 (3.93, 6.07) | <0.001 |
| **GGT, IU/L** | 18 (14, 27) | 21 (15, 30) | 24 (17, 35) | 29 (20, 44) | <0.001 |
| **Serum Iron,u mol/L** | 14.5 (10.9, 19.2) | 15.0 (11.3, 19.2) | 14.9 (11.5, 19.0) | 14.9 (11.5, 18.8) | 0.456 |
| **LDH, IU/L** | 135 (118, 155) | 133 (116, 154) | 134 (117, 153) | 132 (116, 151) | 0.019 |
| **TBil, umol/L** | 10.3 (8.6, 13.7) | 10.3 (8.6, 13.7) | 12.0 (8.6, 13.7) | 10.3 (8.6, 13.7) | 0.265 |
| **Uric acid, umol/L** | 309 (256, 363) | 333 (280, 387) | 348 (297, 405) | 351 (292, 416) | <0.001 |
| **Scr, umol/L** | 73 (62, 88) | 75 (63, 89) | 79 (65, 90) | 78 (63, 90) | 0.049 |
| **Serum Sodium, mmol/L** | 139.58 ± 2.60 | 139.55 ± 2.34 | 139.42 ± 2.29 | 138.71 ± 2.52 | <0.001 |
| **Serum Potassium, mmol/L** | 4.03 ± 0.37 | 4.05 ± 0.36 | 4.07 ± 0.36 | 4.09 ± 0.36 | <0.001 |
| Date are presented as IQR (median) or Mean ± SD; | | | | | |
