# Supplementary material for: The Association Between Cholesterol, High-Density Lipoprotein, and Glucose Index and Mortality in Young and Middle-Aged Adults With Diabetes or Prediabetes: NHANES Data (1999–2018)
Source: Cardiol Res. 2026 Apr 15;17(2):136–48. doi: 10.14740/cr2190 (PMC13094157; doi:10.14740/cr2190)
Supplement: Suppl 5 — Baseline characteristics grouped by age group. [file cr-17-02-136-s005.docx]

**Suppl 5.** Baseline characteristics grouped by age group.

| **Characteristic** | **Categorized by Age (≤ or > 50)** | | |
| --- | --- | --- | --- |
|  | **Overall  N = 14,369** | **Younger  N = 5,678** | **Older  N = 8,691** |
| **Gender, n(%)** |  |  |  |
| Female | 6,597 (45.9%) | 2,380 (41.9%) | 4,217 (48.5%) |
| Male | 7,772 (54.1%) | 3,298 (58.1%) | 4,474 (51.5%) |
| **Race, n(%)** |  |  |  |
| Mexican American | 2,702 (18.8%) | 1,321 (23.3%) | 1,381 (15.9%) |
| Non-Hispanic Black | 2,997 (20.9%) | 1,241 (21.9%) | 1,756 (20.2%) |
| Non-Hispanic White | 6,019 (41.9%) | 1,955 (34.4%) | 4,064 (46.8%) |
| Other Race | 2,651 (18.4%) | 1,161 (20.4%) | 1,490 (17.1%) |
| **Education, n(%)** |  |  |  |
| Less than 9th grade | 2,056 (14.8%) | 525 (10.0%) | 1,531 (17.7%) |
| 9-11th grade | 2,213 (15.9%) | 895 (17.0%) | 1,318 (15.2%) |
| High school graduate or equivalent | 3,290 (23.6%) | 1,244 (23.7%) | 2,046 (23.6%) |
| Some college or Above | 6,369 (45.7%) | 2,594 (49.3%) | 3,775 (43.5%) |
| *Unknown* | *441* | *420* | *21* |
| **Hypertension, n(%)** |  |  |  |
| No | 7,885 (55.0%) | 4,298 (76.0%) | 3,587 (41.3%) |
| Yes | 6,458 (45.0%) | 1,357 (24.0%) | 5,101 (58.7%) |
| *Unknown* | *26* | *23* | *3* |
| **Cerebrovascular disease, n(%)** |  |  |  |
| No | 12,218 (85.0%) | 5,482 (96.5%) | 6,736 (77.5%) |
| Yes | 2,151 (15.0%) | 196 (3.5%) | 1,955 (22.5%) |
| **Smoking status, n(%)** |  |  |  |
| Current | 2,801 (19.9%) | 1,430 (26.6%) | 1,371 (15.8%) |
| Former | 4,084 (29.0%) | 914 (17.0%) | 3,170 (36.5%) |
| Never | 7,178 (51.0%) | 3,040 (56.5%) | 4,138 (47.7%) |
| *Unknown* | *306* | *294* | *12* |
| **Alcohol consumption, n(%)** |  |  |  |
| Heavy | 541 (4.0%) | 319 (6.0%) | 222 (2.7%) |
| Moderate | 4,425 (32.5%) | 2,102 (39.6%) | 2,323 (28.0%) |
| Mild | 6,715 (49.4%) | 2,276 (42.9%) | 4,439 (53.5%) |
| Never | 1,923 (14.1%) | 609 (11.5%) | 1,314 (15.8%) |
| *Unknown* | *765* | *372* | *393* |
| **Diabetes Status, n(%)** |  |  |  |
| Diabetes | 4,189 (29.2%) | 926 (16.3%) | 3,263 (37.5%) |
| Prediabetes | 10,180 (70.8%) | 4,752 (83.7%) | 5,428 (62.5%) |
| **Quartiles of CHG Index, n(%)** |  |  |  |
| Q1(3.07,5.12) | 3,591 (25.0%) | 1,419 (25.0%) | 2,172 (25.0%) |
| Q2(5.12,5.37) | 3,593 (25.0%) | 1,418 (25.0%) | 2,175 (25.0%) |
| Q3(5.37,5.65) | 3,592 (25.0%) | 1,454 (25.6%) | 2,138 (24.6%) |
| Q4(5.65,8.02) | 3,593 (25.0%) | 1,387 (24.4%) | 2,206 (25.4%) |
| **Family poverty income ratio** |  |  |  |
| Mean ± SD | 2.46 ± 1.60 | 2.32 ± 1.61 | 2.55 ± 1.58 |
| Median (Q1, Q3) | 2.03 (1.09, 3.90) | 1.83 (0.96, 3.69) | 2.15 (1.18, 4.05) |
| Min, Max | 0.00, 5.00 | 0.00, 5.00 | 0.00, 5.00 |
| *Unknown* | *1,352* | *462* | *890* |
| **BMI, kg/m^2^** |  |  |  |
| Mean ± SD | 30 ± 7 | 31 ± 8 | 30 ± 6 |
| Median (Q1, Q3) | 29 (25, 34) | 29 (25, 34) | 29 (25, 33) |
| *Unknown* | *249* | *61* | *188* |
| **CHG Index** |  |  |  |
| Mean ± SD | 5.41 ± 0.44 | 5.41 ± 0.44 | 5.41 ± 0.44 |
| Median (Q1, Q3) | 5.37 (5.12, 5.65) | 5.37 (5.12, 5.64) | 5.37 (5.12, 5.65) |
| Min, Max | 3.07, 8.02 | 3.07, 8.02 | 4.00, 7.77 |
