# Supplementary material for: The Association Between Cholesterol, High-Density Lipoprotein, and Glucose Index and Mortality in Young and Middle-Aged Adults With Diabetes or Prediabetes: NHANES Data (1999–2018)
Source: Cardiol Res. 2026 Apr 15;17(2):136–48. doi: 10.14740/cr2190 (PMC13094157; doi:10.14740/cr2190)
Supplement: Suppl 6 — Distribution of mortality outcomes by CHG quartiles in diabetes and prediabetes patients. [file cr-17-02-136-s006.docx]

**Suppl 6.** Distribution of mortality outcomes by CHG quartiles in diabetes and prediabetes patients.


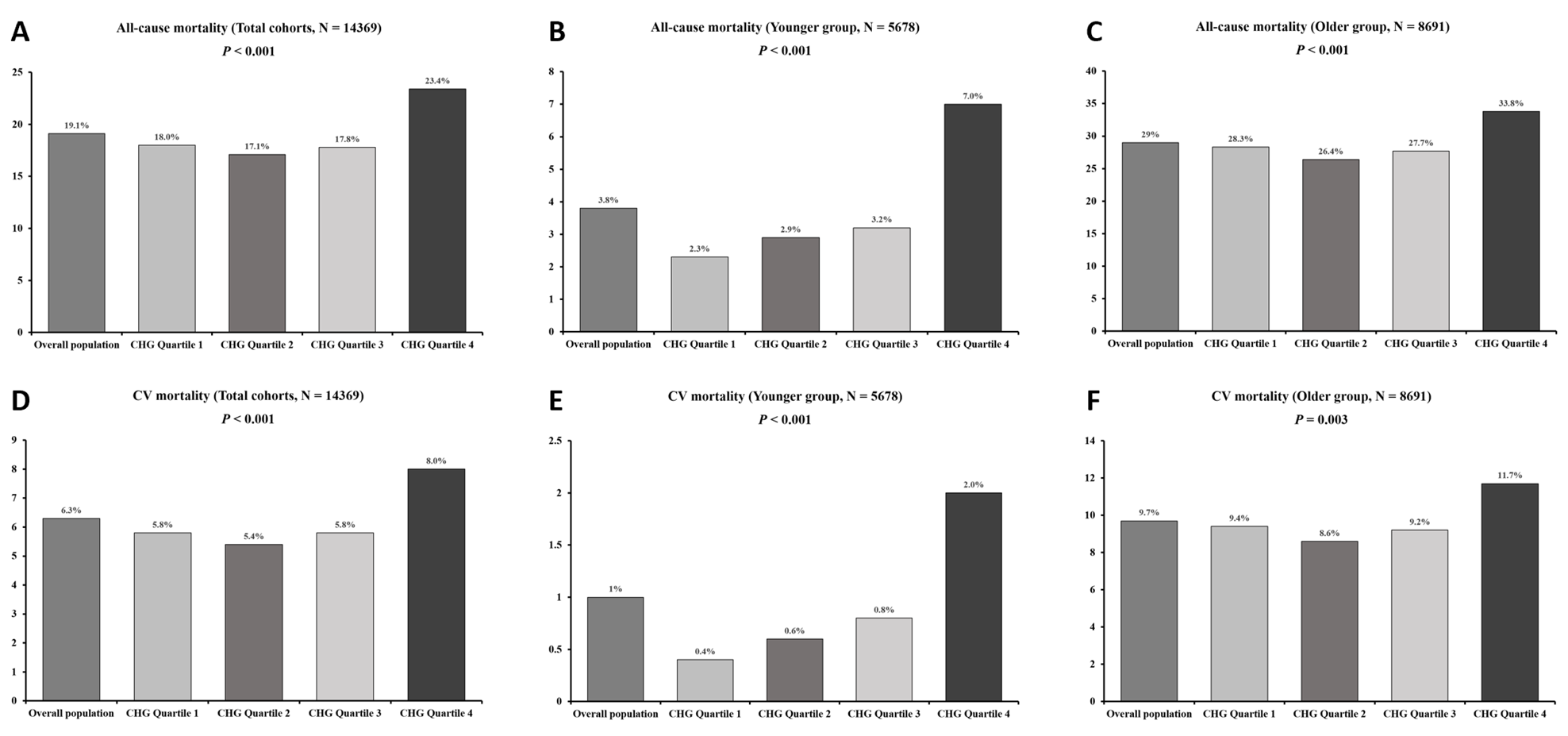


P values from the Fisher’s exact test
