# Supplementary material for: The Association Between Cholesterol, High-Density Lipoprotein, and Glucose Index and Mortality in Young and Middle-Aged Adults With Diabetes or Prediabetes: NHANES Data (1999–2018)
Source: Cardiol Res. 2026 Apr 15;17(2):136–48. doi: 10.14740/cr2190 (PMC13094157; doi:10.14740/cr2190)
Supplement: Suppl 7 — Association between CHG index and all-cause mortality (A) and CV mortality (B) in diabetes and all-cause mortality (C) and CV mortality (D) in pre-diabetes population. [file cr-17-02-136-s007.docx]

**Suppl 7.** Association between CHG index and all-cause mortality (A) and CV mortality (B) in diabetes and all-cause mortality (C) and CV mortality (D) in pre-diabetes population.


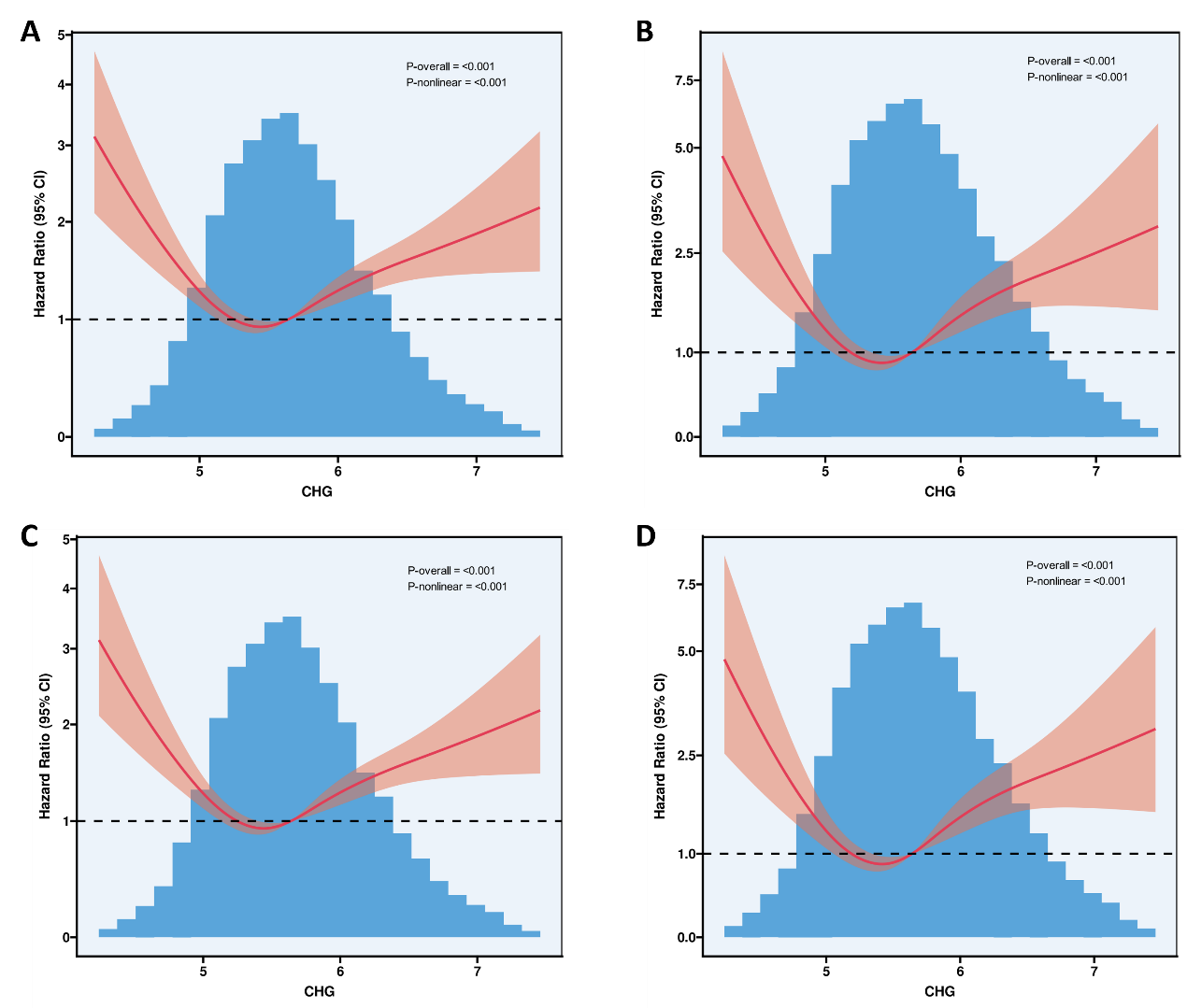


Adjusted for age, gender, race, smoking status, alcohol use, education, hypertension, family income-poverty ratio, Coronary Heart Disease, Congestive Heart Failure, Myocardial infarction, angina pectoris and stroke. The solid line and red area represent the estimated values and their corresponding 95% CIs, respectively.
