# Supplementary material for: The Association Between Cholesterol, High-Density Lipoprotein, and Glucose Index and Mortality in Young and Middle-Aged Adults With Diabetes or Prediabetes: NHANES Data (1999–2018)
Source: Cardiol Res. 2026 Apr 15;17(2):136–48. doi: 10.14740/cr2190 (PMC13094157; doi:10.14740/cr2190)
Supplement: Suppl 8 — Association of CHG index with all-cause mortality and cardiovascular mortality among diabetes or prediabetes population. [file cr-17-02-136-s008.docx]

**Suppl 8.** Association of CHG index with all-cause mortality and cardiovascular mortality among diabetes or prediabetes population.

**
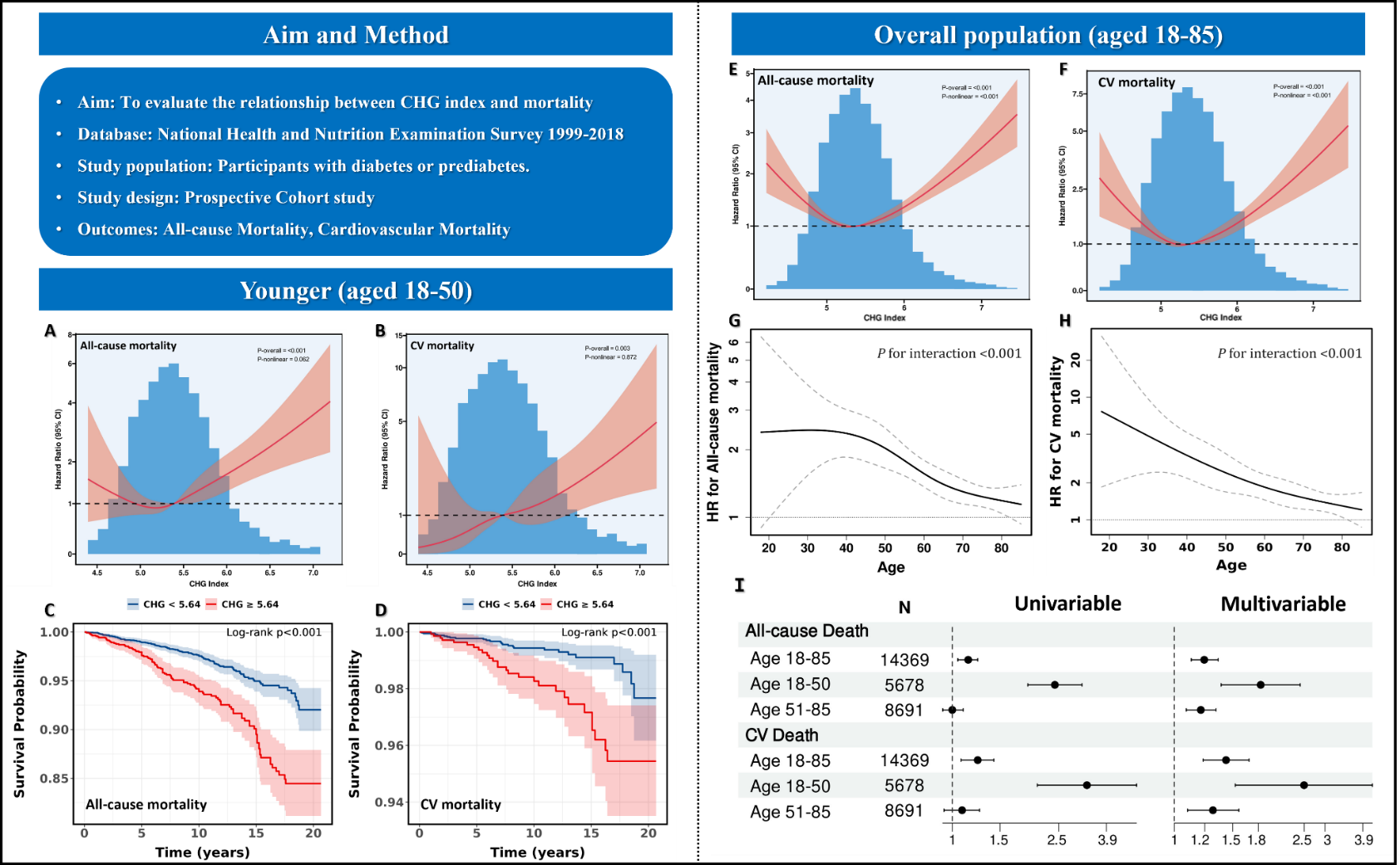
**

**A/B**: RCS models were used to study the link between CHG and mortality among individual aged 18-50 years. **C/D**: Kaplan–Meier survival curve analysis for the all-cause mortality and CV mortality. **E/F**: RCS models analyzed the relationship between CHG and mortality among individual aged 18-85 years. **G/H**: HR for mortality per 1-unit increase in the CHG index (*p* *for interaction < 0.001*). Derived estimates from Cox proportional hazards models, adjusted with restricted cubic splines featuring four knots at 5%, 35%, 65%, and 95%. **I**: Univariable and multivariable Cox proportional hazards regression models were employed to analyze the connection between continuous CHG and mortality.

**A/B/E/F**: The multivariable Cox proportional hazards models' P values were adjusted considering age, gender, race, education level, poverty income ratio, smoking habits, alcohol use, hypertension, and cardiovascular conditions.
