# Supplementary material for: The Association Between Cholesterol, High-Density Lipoprotein, and Glucose Index and Mortality in Young and Middle-Aged Adults With Diabetes or Prediabetes: NHANES Data (1999–2018)
Source: Cardiol Res. 2026 Apr 15;17(2):136–48. doi: 10.14740/cr2190 (PMC13094157; doi:10.14740/cr2190)
Supplement: Suppl 9 — Subgroup analysis of exploring the interaction between CHG index and all-cause mortality outcomes in total cohorts (aged 18 to 85 years). [file cr-17-02-136-s009.docx]

**Suppl 9.** Subgroup analysis of exploring the interaction between CHG index and all-cause mortality outcomes in total cohorts (aged 18 to 85 years)

| Subgroup | N | Crude HR (95% CI) | P value | P for interaction |
| --- | --- | --- | --- | --- |
| Overall | 14369 | 1.25 (1.08-1.44) | 0.003 |  |
| **Age** |  |  |  | <0.001 |
| < 51 | 5678 | 3.26 (2.11-5.04) | <0.001 |  |
| ≥ 51 | 8691 | 1.09 (0.93-1.27) | 0.281 |  |
| **Gender** |  |  |  | 0.55 |
| Female | 6597 | 1.26 (1.01-1.58) | 0.045 |  |
| Male | 7772 | 1.17 (0.97-1.43) | 0.105 |  |
| **Race** |  |  |  | <0.001 |
| Mexican American | 2702 | 3.21 (2.29-4.51) | <0.001 |  |
| Non-Hispanic Black | 2997 | 1.46 (1.09-1.95) | 0.01 |  |
| Non-Hispanic White | 6019 | 1.06 (0.86-1.30) | 0.609 |  |
| Other Race | 2651 | 0.73 (0.40-1.31) | 0.286 |  |
| **Education level** |  |  |  | 0.535 |
| Less than 9th grade | 2056 | 1.23 (0.90-1.68) | 0.198 |  |
| 9-11th grade | 2213 | 0.93 (0.68-1.29) | 0.683 |  |
| High school graduate or equivalent | 3290 | 1.23 (0.91-1.67) | 0.185 |  |
| Some college or Above | 6369 | 1.21 (0.93-1.57) | 0.151 |  |
| **Cerebrovascular disease** |  |  |  | <0.001 |
| No | 12218 | 1.43 (1.18-1.72) | <0.001 |  |
| Yes | 2151 | 0.86 (0.69-1.08) | 0.203 |  |
| **Smoking status** |  |  |  | 0.16 |
| Current | 2801 | 0.86 (0.60-1.25) | 0.441 |  |
| Former | 4084 | 1.24 (0.99-1.57) | 0.066 |  |
| Never | 7178 | 1.30 (1.04-1.62) | 0.02 |  |
| **Hypertension** |  |  |  | 0.001 |
| No | 7885 | 1.65 (1.29-2.10) | <0.001 |  |
| Yes | 6458 | 0.99 (0.83-1.18) | 0.896 |  |
| **Alcohol consumption** |  |  |  | 0.161 |
| Heavy | 541 | 0.16 (0.01-3.32) | 0.235 |  |
| Moderate | 4425 | 1.06 (0.79-1.42) | 0.719 |  |
| Mild | 6715 | 1.24 (1.02-1.50) | 0.03 |  |
| Never | 1923 | 1.47 (1.05-2.06) | 0.024 |  |
