# Supplementary material for: The Association Between Cholesterol, High-Density Lipoprotein, and Glucose Index and Mortality in Young and Middle-Aged Adults With Diabetes or Prediabetes: NHANES Data (1999–2018)
Source: Cardiol Res. 2026 Apr 15;17(2):136–48. doi: 10.14740/cr2190 (PMC13094157; doi:10.14740/cr2190)
Supplement: Suppl 10 — Subgroup analysis of exploring the interaction between CHG index and CV-related mortality outcomes in total cohorts (aged 18 to 85 years). [file cr-17-02-136-s010.docx]

**Suppl 10.** Subgroup analysis of exploring the interaction between CHG index and CV-related mortality outcomes in total cohorts (aged 18 to 85 years)

| Subgroup | N | Crude HR (95% CI) | P value | P for interaction |
| --- | --- | --- | --- | --- |
| Overall | 14369 | 1.15 (1.05-1.25) | 0.001 |  |
| **Age** |  |  |  | <0.001 |
| < 51 | 5678 | 2.46 (1.94-3.12) | <0.001 |  |
| ≥ 51 | 8691 | 1.00 (0.92-1.10) | 0.929 |  |
| **Gender** |  |  |  | 0.015 |
| Female | 6597 | 1.25 (1.10-1.42) | 0.001 |  |
| Male | 7772 | 1.03 (0.92-1.15) | 0.637 |  |
| **Race** |  |  |  | <0.001 |
| Mexican American | 2702 | 1.78 (1.45-2.19) | <0.001 |  |
| Non-Hispanic Black | 2997 | 1.38 (1.15-1.64) | <0.001 |  |
| Non-Hispanic White | 6019 | 1.02 (0.90-1.15) | 0.756 |  |
| Other Race | 2651 | 1.09 (0.82-1.46) | 0.544 |  |
| **Education level** |  |  |  | 0.041 |
| Less than 9th grade | 2056 | 0.89 (0.74-1.07) | 0.213 |  |
| 9-11th grade | 2213 | 0.92 (0.76-1.11) | 0.389 |  |
| High school graduate or equivalent | 3290 | 1.18 (0.99-1.41) | 0.059 |  |
| Some college or Above | 6369 | 1.14 (0.98-1.33) | 0.093 |  |
| **Cerebrovascular disease** |  |  |  | <0.001 |
| No | 12218 | 1.23 (1.11-1.36) | <0.001 |  |
| Yes | 2151 | 0.86 (0.74-0.99) | 0.037 |  |
| **Smoking status** |  |  |  | 0.202 |
| Current | 2801 | 1.09 (0.91-1.31) | 0.356 |  |
| Former | 4084 | 0.99 (0.86-1.14) | 0.896 |  |
| Never | 7178 | 1.17 (1.02-1.34) | 0.025 |  |
| **Hypertension** |  |  |  | <0.001 |
| No | 7885 | 1.34 (1.17-1.53) | <0.001 |  |
| Yes | 6458 | 0.96 (0.86-1.07) | 0.501 |  |
| **Alcohol consumption** |  |  |  | 0.06 |
| Heavy | 541 | 0.46 (0.13-1.64) | 0.232 |  |
| Moderate | 4425 | 1.01 (0.85-1.20) | 0.904 |  |
| Mild | 6715 | 1.15 (1.02-1.28) | 0.017 |  |
| Never | 1923 | 1.36 (1.10-1.66) | 0.004 |  |
