# Supplementary material for: The Association Between Cholesterol, High-Density Lipoprotein, and Glucose Index and Mortality in Young and Middle-Aged Adults With Diabetes or Prediabetes: NHANES Data (1999–2018)
Source: Cardiol Res. 2026 Apr 15;17(2):136–48. doi: 10.14740/cr2190 (PMC13094157; doi:10.14740/cr2190)
Supplement: Suppl 11 — HRs (95% CIs) for mortality according to the CHG index quartiles in total cohorts (aged 18 to 85 years). [file cr-17-02-136-s011.docx]

**Suppl 11.** HRs (95% CIs) for mortality according to the CHG index quartiles in total cohorts (aged 18 to 85 years)

| **Characteristic** | **Model 1** | | | **Model 2** | | | **Model 3** | | |
| --- | --- | --- | --- | --- | --- | --- | --- | --- | --- |
|  | **HR** | **95% CI** | **p-value** | **HR** | **95% CI** | **p-value** | **HR** | **95% CI** | **p-value** |
| **All-cause mortality** |  |  |  |  |  |  |  |  |  |
| **CHG index (continuous)** | | | | | | | | | |
|  | 1.15 | 1.05-1.25 | 0.001 | 1.26 | 1.15-1.38 | <0.001 | 1.24 | 1.13-1.36 | <0.001 |
| **CHG index** |  |  |  |  |  |  |  |  |  |
| Q1 | 1 (Reference) | | | 1 (Reference) | | | 1 (Reference) | | |
| Q2 | 0.86 | 0.77-0.96 | 0.009 | 0.84 | 0.75-0.94 | 0.003 | 0.85 | 0.76-0.95 | 0.004 |
| Q3 | 0.82 | 0.73-0.91 | <0.001 | 0.82 | 0.73-0.92 | <0.001 | 0.81 | 0.72-0.91 | <0.001 |
| Q4 | 1.08 | 0.97-1.19 | 0.162 | 1.12 | 1.00-1.25 | 0.044 | 1.08 | 0.97-1.21 | 0.160 |
| **CV mortality** |  |  |  |  |  |  |  |  |  |
| **CHG index (continuous)** | | | | | | | | | |
|  | **1.25** | **1.08-1.44** | **0.003** | **1.43** | **1.22-1.67** | **<0.001** | **1.41** | **1.21-1.65** | **<0.001** |
| **CHG index** |  |  |  |  |  |  |  |  |  |
| Q1 | 1 (Reference) | | | 1 (Reference) | | | 1 (Reference) | | |
| Q2 | 0.84 | 0.69-1.02 | 0.080 | 0.82 | 0.67-1.00 | 0.046 | 0.84 | 0.69-1.03 | 0.089 |
| Q3 | 0.82 | 0.67-0.99 | 0.039 | 0.81 | 0.66-0.99 | 0.035 | 0.81 | 0.67-0.99 | 0.042 |
| Q4 | **1.13** | **0.95-1.35** | **0.178** | **1.20** | **1.00-1.45** | **0.055** | 1.18 | 0.98-1.42 | 0.088 |

| HR = Hazard Ratio, CI = Confidence Interval |
| --- |
| Model 1 : no covariates were adjusted  Model 2 : Age, Race, Gender, Education level, and family income-poverty ratio, Model 3 : Model 2 + Hypertension, CVD (Coronary Heart Disease, Congestive Heart Failure, Myocardial infarction, angina pectoris, stroke) ,Smoking status, and Alcohol consumption |
