# Supplementary material for: The Association Between Cholesterol, High-Density Lipoprotein, and Glucose Index and Mortality in Young and Middle-Aged Adults With Diabetes or Prediabetes: NHANES Data (1999–2018)
Source: Cardiol Res. 2026 Apr 15;17(2):136–48. doi: 10.14740/cr2190 (PMC13094157; doi:10.14740/cr2190)
Supplement: Suppl 13 — Sensitivity analysis of CHG index and mortality outcomes in patients after excluding patients died within the first 24-month follow-up. [file cr-17-02-136-s013.docx]

**Suppl 13.** Sensitivity analysis of CHG index and mortality outcomes in patients after excluding patients died within the first 24-month follow-up

|  | All-cause mortality | | Cardiovascular mortality | |
| --- | --- | --- | --- | --- |
| CHG quartiles | HR (95% CI) | *P* | HR (95% CI) | *P* |
| **Overall** | | | | |
| Q1 | Ref |  | Ref |  |
| Q2 | 0.82 (0.72, 0.94) | 0.004 | 0.76 (0.60, 0.96) | 0.020 |
| Q3 | 0.78 (0.68, 0.89) | <0.001 | 0.71 (0.56, 0.89) | 0.004 |
| Q4 | 1.07 (0.94, 1.22) | 0.293 | 1.01 (0.80, 1.27) | 0.940 |
| **Younger** | | | | |
| Q1 | Ref |  | Ref |  |
| Q2 | 0.94 (0.56, 1.55) | 0.794 | 0.96 (0.29, 3.17) | 0.943 |
| Q3 | 0.93 (0.56, 1.52) | 0.765 | 1.26 (0.42, 3.79) | 0.687 |
| Q4 | **1.61 (1.03, 2.52)** | **0.037** | 2.46 (0.89, 6.77) | 0.083 |
| **Older** |  |  |  |  |
| Q1 | Ref |  | Ref |  |
| Q2 | 0.85 (0.74, 0.96) | 0.013 | 0.80 (0.64, 1.01) | 0.943 |
| Q3 | 0.80 (0.70, 0.91) | <0.001 | 0.76 (0.60, 0.95) | 0.018 |
| Q4 | 1.08 (0.95, 1.23) | 0.241 | 1.17 (0.94, 1.45) | 0.173 |

Models were adjusted for age, sex, race, education level, poverty income ratio, smoking status, alcohol consumption, hypertension and CVD.
