# Supplementary material for: The Association Between Cholesterol, High-Density Lipoprotein, and Glucose Index and Mortality in Young and Middle-Aged Adults With Diabetes or Prediabetes: NHANES Data (1999–2018)
Source: Cardiol Res. 2026 Apr 15;17(2):136–48. doi: 10.14740/cr2190 (PMC13094157; doi:10.14740/cr2190)
Supplement: Suppl 14 — Sensitivity analysis of CHG index and mortality outcomes in patients after excluding patients after excluding patients with CVD. [file cr-17-02-136-s014.docx]

**Suppl 14.** Sensitivity analysis of CHG index and mortality outcomes in patients after excluding patients after excluding patients with CVD

|  | All-cause mortality | | Cardiovascular mortality | |
| --- | --- | --- | --- | --- |
| CHG quartiles | HR (95% CI) | *P* | HR (95% CI) | *P* |
| **Overall** | | | | |
| Q1 | Ref |  | Ref |  |
| Q2 | 0.92 (0.79, 1.07) | 0.288 | 0.85 (0.64, 1.14) | 0.282 |
| Q3 | 0.85 (0.73, 1.00) | 0.044 | 0.90 (0.67, 1.19) | 0.456 |
| Q4 | 1.15 (0.99, 1.33) | 0.075 | 1.25 (0.95, 1.66) | 0.940 |
| **Younger** | | | | |
| Q1 | Ref |  | Ref |  |
| Q2 | 0.97 (0.57, 1.66) | 0.923 | 1.43 (0.40, 5.07) | 0.579 |
| Q3 | 1.01 (0.60, 1.70) | 0.957 | 2.18 (0.68, 6.94) | 0.189 |
| Q4 | **1.78 (1.10, 2.86)** | **0.018** | **4.85(1.67, 14.04)** | **0.004** |
| **Older** |  |  |  |  |
| Q1 | Ref |  | Ref |  |
| Q2 | 0.94 (0.80, 1.09) | 0.407 | 0.94 (0.70, 1.26) | 0.667 |
| Q3 | 0.85 (0.73, 1.00) | 0.048 | 0.93 (0.70, 1.25) | 0.642 |
| Q4 | 1.13 (0.97, 1.31) | 0.130 | 1.37 (1.04, 1.82) | 0.027 |

Models were adjusted for age, sex, race, education level, poverty income ratio, smoking status, alcohol consumption, and hypertension
