# Supplementary material for: The Association Between Cholesterol, High-Density Lipoprotein, and Glucose Index and Mortality in Young and Middle-Aged Adults With Diabetes or Prediabetes: NHANES Data (1999–2018)
Source: Cardiol Res. 2026 Apr 15;17(2):136–48. doi: 10.14740/cr2190 (PMC13094157; doi:10.14740/cr2190)
Supplement: Suppl 15 — Subgroup analysis of exploring the interaction between CHG index (quartile 4) and all-cause mortality in population aged 18 to 50 years. [file cr-17-02-136-s015.docx]

**Suppl 15.** Subgroup analysis of exploring the interaction between CHG index (Quartile 4) and all-cause mortality in population aged 18 to 50 years

| Subgroup | < 5.64 | ≥ 5.64 | HR (95% CI) | P value | P for interaction |
| --- | --- | --- | --- | --- | --- |
| **Overall** | 117/4,251 (2.8) | 100/1,427 (7.0) | 2.32 (1.77-3.03) | <0.001 |  |
| **Age** |  |  |  |  | 0.169 |
| < 35 | 20/1,871 (1.1) | 15/412 (3.6) | 3.09 (1.58-6.03) | 0.001 |  |
| ≥ 35 | 97/2,380 (4.1) | 85/1,015 (8.4) | 1.85 (1.38-2.48) | <0.001 |  |
| **Education level** |  |  |  |  | 0.785 |
| Less than 9th grade | 11/344 (3.2) | 11/181 (6.1) | 1.73 (0.75-3.98) | 0.201 |  |
| 9-11th grade | 28/641 (4.4) | 21/254 (8.3) | 1.83 (1.04-3.22) | 0.037 |  |
| High school graduate or equivalent | 26/885 (2.9) | 30/359 (8.4) | 2.47 (1.46-4.19) | 0.001 |  |
| Some college or Above | 48/2,008 (2.4) | 36/586 (6.1) | 2.35 (1.53-3.63) | <0.001 |  |
| **Race** |  |  |  |  | 0.988 |
| Mexican American | 17/949 (1.8) | 17/372 (4.6) | 2.33 (1.19-4.56) | 0.014 |  |
| Non-Hispanic Black | 38/1,017 (3.7) | 22/224 (9.8) | 2.42 (1.43-4.09) | 0.001 |  |
| Non-Hispanic White | 54/1,405 (3.8) | 55/550 (10.0) | 2.46 (1.69-3.58) | <0.001 |  |
| Other Race | 8/880 (0.9) | 6/281 (2.1) | 1.89 (0.65-5.47) | 0.240 |  |
| **Cerebrovascular disease** |  |  |  |  | 0.709 |
| No | 105/4,137 (2.5) | 82/1,345 (6.1) | 2.17 (1.62-2.90) | <0.001 |  |
| Yes | 12/114 (10.5) | 18/82 (22.0) | 1.82 (0.87-3.78) | 0.110 |  |
| **Smoking status** |  |  |  |  | 0.900 |
| Current | 58/984 (5.9) | 55/446 (12.3) | 2.03 (1.40-2.93) | <0.001 |  |
| Former | 20/661 (3.0) | 16/253 (6.3) | 1.94 (1.00-3.74) | 0.049 |  |
| Never | 35/2,351 (1.5) | 28/689 (4.1) | 2.30 (1.40-3.79) | 0.001 |  |
| **Hypertension** |  |  |  |  | 0.943 |
| No | 69/3,338 (2.1) | 47/960 (4.9) | 2.01 (1.39-2.92) | <0.001 |  |
| Yes | 48/893 (5.4) | 53/464 (11.4) | 2.04 (1.38-3.02) | <0.001 |  |
| **Alcohol consumption** |  |  |  |  | 0.675 |
| Heavy | 3/252 (1.2) | 1/67 (1.5) | 2.57 (0.25-25.97) | 0.424 |  |
| Moderate | 49/1,472 (3.3) | 43/630 (6.8) | 1.93 (1.28-2.91) | 0.002 |  |
| Mild | 52/1,743 (3.0) | 44/533 (8.3) | 2.51 (1.68-3.75) | <0.001 |  |
| Never | 9/471 (1.9) | 11/138 (8.0) | 3.16 (1.30-7.69) | 0.011 |  |
| no. of events / total no. (%) | | | | | |
