# Supplementary material for: The Association Between Cholesterol, High-Density Lipoprotein, and Glucose Index and Mortality in Young and Middle-Aged Adults With Diabetes or Prediabetes: NHANES Data (1999–2018)
Source: Cardiol Res. 2026 Apr 15;17(2):136–48. doi: 10.14740/cr2190 (PMC13094157; doi:10.14740/cr2190)
Supplement: Suppl 16 — Subgroup analysis of exploring the interaction between CHG index (quartile 4) and CV mortality in population aged 18 to 50 years. [file cr-17-02-136-s016.docx]

**Suppl 16.** Subgroup analysis of exploring the interaction between CHG index (Quartile 4) and CV mortality in population aged 18 to 50 years

| Subgroup | < 5.64 | ≥ 5.64 | HR (95% CI) | P value | P for interaction |
| --- | --- | --- | --- | --- | --- |
| **Overall** | 26/4,251 (0.6) | 28/1,427 (2.0) | 2.91 (1.70-4.96) | <0.001 |  |
| **Gender** |  |  |  |  | 0.753 |
| Female | 7/1,949 (0.4) | 4/431 (0.9) | 2.34 (0.68-8.00) | 0.175 |  |
| Male | 19/2,302 (0.8) | 24/996 (2.4) | 2.76 (1.51-5.03) | 0.001 |  |
| **Age** |  |  |  |  | 0.136 |
| < 35 | 4/1,871 (0.2) | 6/412 (1.5) | 6.24 (1.76-22.12) | 0.005 |  |
| ≥ 35 | 22/2,380 (0.9) | 22/1,015 (2.2) | 2.10 (1.16-3.80) | 0.014 |  |
| **Race** |  |  |  |  | 0.296 |
| Mexican American | 3/949 (0.3) | 7/372 (1.9) | 5.43 (1.40-21.02) | 0.014 |  |
| Non-Hispanic Black | 13/1,017 (1.3) | 7/224 (3.1) | 2.25 (0.90-5.67) | 0.084 |  |
| Non-Hispanic White | 10/1,405 (0.7) | 12/550 (2.2) | 2.86 (1.23-6.63) | 0.014 |  |
| Other Race | 0/880 (0.0) | 2/281 (0.7) | NA | 0.999 |  |
| **Education level** |  |  |  |  | 0.114 |
| Less than 9th grade | 2/344 (0.6) | 1/181 (0.6) | 0.83 (0.08-9.22) | 0.882 |  |
| 9-11th grade | 10/641 (1.6) | 5/254 (2.0) | 1.22 (0.42-3.56) | 0.721 |  |
| High school graduate or equivalent | 6/885 (0.7) | 8/359 (2.2) | 2.88 (1.00-8.32) | 0.051 |  |
| Some college or Above | 8/2,008 (0.4) | 14/586 (2.4) | 5.59 (2.34-13.35) | <0.001 |  |
| **Cerebrovascular disease** |  |  |  |  | 0.337 |
| No | 21/4,137 (0.5) | 22/1,345 (1.6) | 2.88 (1.58-5.25) | 0.001 |  |
| Yes | 5/114 (4.4) | 6/82 (7.3) | 1.45 (0.44-4.77) | 0.540 |  |
| **Smoking status** |  |  |  |  | 0.539 |
| Current | 13/984 (1.3) | 13/446 (2.9) | 2.12 (0.98-4.57) | 0.056 |  |
| Former | 5/661 (0.8) | 4/253 (1.6) | 1.90 (0.51-7.07) | 0.341 |  |
| Never | 8/2,351 (0.3) | 11/689 (1.6) | 4.08 (1.64-10.16) | 0.002 |  |
| **Hypertension** |  |  |  |  | 0.081 |
| No | 11/3,338 (0.3) | 15/960 (1.6) | 3.98 (1.82-8.69) | 0.001 |  |
| Yes | 15/893 (1.7) | 13/464 (2.8) | 1.59 (0.76-3.35) | 0.2 |  |
| **Alcohol consumption** |  |  |  |  | 0.912 |
| Heavy | 0/252 (0.0) | 0/67 (0.0) | NA |  |  |
| Moderate | 13/1,472 (0.9) | 13/630 (2.1) | 2.19 (1.02-4.74) | 0.045 |  |
| Mild | 10/1,743 (0.6) | 11/533 (2.1) | 3.25 (1.38-7.67) | 0.007 |  |
| Never | 3/471 (0.6) | 4/138 (2.9) | 3.81 (0.85-17.16) | 0.081 |  |
